# Evidence that the Human Pathogenic Fungus *Cryptococcus neoformans* var. *grubii* May Have Evolved in Africa

**DOI:** 10.1371/journal.pone.0019688

**Published:** 2011-05-11

**Authors:** Anastasia P. Litvintseva, Ignazio Carbone, Jenny Rossouw, Rameshwari Thakur, Nelesh P. Govender, Thomas G. Mitchell

**Affiliations:** 1 Department of Molecular Genetics and Microbiology, Duke University Medical Center, Durham, North Carolina, United States of America; 2 Center for Integrated Fungal Research, Department of Plant Pathology, North Carolina State University, Raleigh, North Carolina, United States of America; 3 Special Bacterial Pathogens Reference Unit, National Institute for Communicable Diseases, Johannesburg, Republic of South Africa; 4 Mycology Department, National Health Laboratory, Ministry of Health, Gaborone, Botswana; 5 Mycology Reference Unit, National Institute for Communicable Diseases, Johannesburg, Republic of South Africa; University of Minnesota, United States of America

## Abstract

Most of the species of fungi that cause disease in mammals, including *Cryptococcus neoformans* var. *grubii* (serotype A), are exogenous and non-contagious. *Cryptococcus neoformans* var. *grubii* is associated worldwide with avian and arboreal habitats. This airborne, opportunistic pathogen is profoundly neurotropic and the leading cause of fungal meningitis. Patients with HIV/AIDS have been ravaged by cryptococcosis – an estimated one million new cases occur each year, and mortality approaches 50%. Using phylogenetic and population genetic analyses, we present evidence that *C. neoformans* var. *grubii* may have evolved from a diverse population in southern Africa. Our ecological studies support the hypothesis that a few of these strains acquired a new environmental reservoir, the excreta of feral pigeons (*Columba livia*), and were globally dispersed by the migration of birds and humans. This investigation also discovered a novel arboreal reservoir for highly diverse strains of *C. neoformans* var. *grubii* that are restricted to southern Africa, the mopane tree (*Colophospermum mopane*). This finding may have significant public health implications because these primal strains have optimal potential for evolution and because mopane trees contribute to the local economy as a source of timber, folkloric remedies and the edible mopane worm.

## Introduction

The inexorably neurotropic, environmental yeast, *Cryptococcus neoformans* var. *grubii*, is an opportunistic human pathogen and the leading cause of fungal meningoencephalitis [1], [2]. Most cases of cryptococcal disease occur in patients who are immunocompromised. Cryptococcosis is an AIDS-defining illness [3], and in sub-Saharan Africa, an estimated one million new cases of cryptococcal meningitis occur annually with mortality rates that may exceed 50% [4]. Patients in North America and Europe have better access to treatment of both HIV and cryptococcosis, and the incidence of cryptococcosis is much lower, but mortality still approaches 40% [5], [6]. Cryptococcosis can be caused by either of two species, *C. neoformans*, which is characterized by haploid isolates with the A or D capsular serotype, as well as AD hybrids, or *C. gattii*, traditionally denoted by serotype B or C. However, more than 90% of infections worldwide are due to haploid strains of *C. neoformans* var. *grubii*, which possess the serotype A capsular epitope [2].

In the environment, strains of *C. neoformans* var. *grubii* are commonly associated with decayed wood, soil and pigeon excreta, and infections are acquired by inhaling airborne yeasts or basidiospores [2], [7], [8]. Pigeons and most other birds do not acquire cryptococcosis because the avian body temperature is too high to support the growth of cryptococcal cells; however, the excrement of columbine birds is a natural enrichment medium for *C. neoformans* var. *grubii*, and the birds serve as vectors to disseminate the yeasts [9]. Recent genotypic analyses of global clinical and environmental isolates of *C. neoformans* var. *grubii* identified three genetic subpopulations, VNI, VNII, and VNB. Isolates of VNI are the most ubiquitous and prevalent, causing the majority of worldwide cases of cryptococcosis. Strains of VNII are globally distributed but rare. VNB strains are highly diverse and apparently restricted geographically to southern Africa [10]. Our previous study and this investigation indicate that many VNI strains are also endemic to southern Africa [10].

The majority of natural isolates are haploid, but they possess one of two mating type alleles, α or **a**, and in the laboratory, strains of opposite mating type are capable of sexual reproduction [11]. However, 99.9% of the cosmopolitan isolates of VNI and all known isolates of VNII possess only the α mating type (*MAT*α), and this dominance of a single mating type minimizes the possibility of conventional sexual reproduction in nature. A possible alternative, mating between isolates of the same mating type, has been demonstrated [12]. Conversely, 22% of the VNB strains and approximately 1 to 4% of the African VNI strains carry the *MAT* **a** mating type allele, reproduce sexually with *MAT*α strains in the laboratory and generate fertile progeny [13]. Because of the unusually high genetic diversity among African VNI and VNB strains, we hypothesized that southern Africa may harbor the ancestral populations of *C. neoformans* var. *grubii*. Here, we applied methods of population genetics and phylogenetics to analyze the population structures and demographic histories of both global and African strains. The data provide evidence that the global isolates of *C. neoformans* var. *grubii* originated in Africa.

## Results

### In southern Africa, strains of *C. neoformans* var. *grubii* are associated with native trees and pigeon excreta

The remarkable genetic diversity of strains in southern Africa suggests that this region may represent the evolutionary origin of *C. neoformans* var. *grubii*. To test this hypothesis, we obtained 273 *C. neoformans* var. *grubii* isolates from a variety of environmental niches in southern Africa. Twenty-two sites produced positive isolates of *C. neoformans* var. *grubii* (Table S1, Fig. S1). We sampled many putative niches, but the most frequently positive sites were trees or soil at the base of trees (16 sites were positive for *C. neoformans* var. *grubii*; Table S1) and avian, usually pigeon feces (six sites were positive for *C. neoformans* var. *grubii*; Table S1). The highest number of isolates was obtained from decayed hollows of the endemic southern African tree, *Colophospermum mopane*. Ten of 31 (32%) sampled mopane trees were colonized by *C. neoformans* var. *grubii* (Table S1). No isolates of serotype D or AD hybrids were found.

### Environmental isolates from Africa are haploid, and arboreal strains may possess the rare *MAT*a mating type allele

Previous studies of non-African isolates of *C. neoformans* var. *grubii* indicated that approximately 10% of clinical and environmental strains are diploid [14], [15]. We used flow cytometry to measure the relative DNA content of 46 representative strains of *C. neoformans* var. *grubii* isolated from the African environment (two strains per site), and all tested strains were haploid. In addition, our previous analysis indicated that approximately 10% of clinical strains from Botswana possessed the rare *MAT* **a** mating type allele [13]. Using PCR primers specific for *MAT* **a** and *MATα* alleles, we determined that all strains isolated from pigeon feces possessed the *MATα* mating type allele; however, one of 16 arboreal sites was colonized by strains with the *MAT* **a** mating type. Specifically, 10 isolates with identical genotypes and the *MAT* **a** mating type were isolated from the Tu422 site, which was associated with a mopane tree (Table S1). When these *MAT* **a** isolates were cultured with tester strains possessing the *MATα* allele [14], [15], they successfully mated and produced basidiospores (data not shown).

### Multilocus analysis and population genetics indicate that global strains of *C. neoformans* var. *grubii* are associated with pigeon excreta and that African strains are associated with endemic African trees

To study the origin of *C. neoformans* var. *grubii*, 58 environmental and 59 clinical strains from Africa were genotyped by multilocus sequence typing (MLST) using eight loci, including seven MLST consensus loci [16] and the *TEF1* locus, which is useful in differentiating VNB strains [10]. These genotypes were compared with 25 representative isolates from the global population (Table S2).

The following criteria were used to select the representative strains for this analysis (Table S2): (i) We included all available clinical strains that were isolated in 2006 and 2007 from Botswana and the adjoining South African province of Limpopo. (ii) The environmental isolates were pre-screened using the four most variable MLST loci (*GPD1*, *PLB*, *SOD1* and *TEF1*) to detect and remove redundant, clonal isolates (data not shown). Then, each unique, polymorphic environmental isolate was genotyped using the complete panel of eight MLST loci. (iii) Representative strains from the global population were selected from our previous analysis of 102 *C. neoformans* var. *grubii* isolates from 15 countries using twelve unlinked MLST markers [10]. (iv) To ensure an unbiased, comprehensive selection of strains, we included every unique MLST genotype and at least one representative from each country.

**Figure 1:**
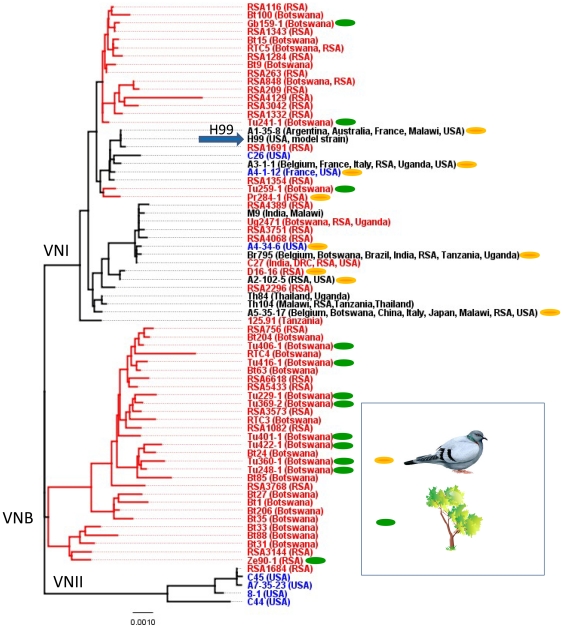
This unrooted dendrogram depicts the genetic relationships of MLST genotypes among isolates of *C. neoformans* var. *grubii*. DNA sequences of eight loci were concatenated (totaling 4,443 base pairs) and analyzed with the neighbor joining method using uncorrected (“p”) genetic distances. The three major subpopulations or divergent clades of *C. neoformans* var. *grubii*, VNI, VNII, and VNB, are apparent. Isolates are clone-corrected (i.e., only one strain of each unique genotype is included). For each genotype, the country or countries of origin is/are shown in parentheses (RSA, Republic of South Africa; DCR, Democratic Republic of Congo). Strains that are unique to Africa are labeled in red, strains that are not found in Africa are labeled in blue, strains that are isolated from Africa and elsewhere are labeled in black. The ecological origin of each strain is indicated by colored ellipses. Green indicates that the strain was isolated from trees and/or soil at the base of trees, and an orange ellipse denotes that the strain was isolated from pigeon feces or soil contaminated with avian feces. Strains without an accompanying green or orange ellipse are clinical isolates that have not been yet been isolated from the environment. In addition, regardless of the ecological source of an isolate, most of the strains or their clones have been isolated from patients. Model strain H99, which is the subject of most molecular studies of *C. neoformans*, is labeled with arrow [62]. Refer to Table S2 for details about each isolate.

**Figure 2:**
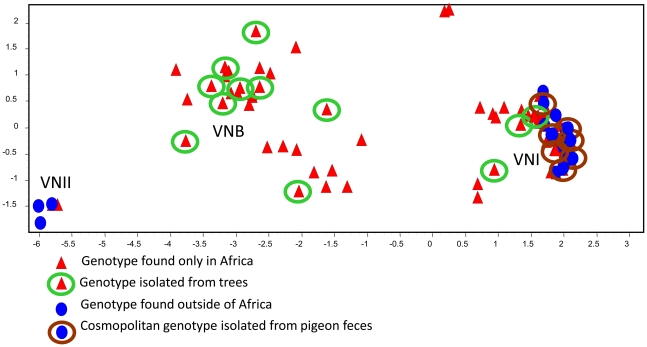
The genetic relationships among 72 MLST genotypes are visualized by Principal Component analysis (PCA). Each symbol represents a genotype with a unique eight-digit allelic profile (re Fig. 4). Red triangles represent genotypes of strains that are endemic to Africa, and blue circles represent genotypes of global strains. Genotypes associated with African trees are enclosed in green circles, and genotypes associated with pigeon excreta are enclosed in brown circles. Genotypes without circles represent clinical strains that to date have not been isolated from the environment.

The genetic relationships among the genotypes were evaluated by pairwise distance (Fig. 1) and principal component (Fig. 2) analyses. Both methods indicated that only a few genotypes are globally distributed, and the majority of genotypes are apparently confined to southern Africa. In addition, the analyses detected a significant correlation (*p*<0.001, Fisher's exact test) between the MLST genotypes and the ecological sources of the isolates: strains with cosmopolitan genotypes were associated with pigeon excreta, and the more diverse African genotypes were found in trees or soil from the base of the trees (Figs. 1 and 2).

Strains of *C. neoformans* var. *grubii* with certain identical MLST genotypes were found globally and isolated from pigeon habitats in Africa, North America and Europe [8]. However, the MLST genotypes of strains isolated from African trees were found nowhere else. Clinical isolates of *C. neoformans* var. *grubii* that were obtained from patients who were unlikely to have traveled beyond sub-Saharan Africa included strains with global as well as exclusively African genotypes. Figures 1 and 2 suggest that southern Africa harbors two ecologically and geographically isolated subpopulations of *C. neoformans* var. *grubii*: (i) an endemic arboreal population, which is confined to rural areas and associated with native trees, and (ii) an avian, coprophilic population, which is restricted to urban locations and associated with columbine excreta.

This conclusion was supported by Wright's fixation index (*F_st_*) [17], which we calculated to estimate the level of genetic interaction between avian and arboreal African populations of *C. neoformans* var. *grubii.* The *F_st_* was significantly higher than 0 (*F_st_* = 0.1, *p*<0.001), indicating restricted genetic exchange between populations associated with these ecological niches in Africa. However, a comparison of global and African strains associated with pigeons yielded an *F_st_* of 0.04, which was not significantly different from 0 (*p* = 0.11), indicating high gene flow among isolates from pigeons regardless of their geographic location.

One possible explanation for the limited genetic exchange between avian coprophilic and arboreal populations in Africa may be that strains from trees are unable to grow in pigeon excreta (or vice versa). To test this possibility, strains of *C. neoformans* var. *grubii* with different genotypes were cultured on media prepared with boiled pigeon excreta or mopane bark. All strains grew equally well on both arboreal and avian fecal media (Fig. S2), indicating that at least under laboratory conditions, there was no evidence of substrate specificity among strains from these different niches.

The limited genetic exchange between sympatric arboreal and avian populations may also be attributed to (i) mating incompatibility and/or (ii) spatial segregation. The first possibility is unlikely because arboreal VNB and avian VNI strains were able to mate in the laboratory and produce viable basidiospores (data not shown). However, geographic and ecological isolation provides a plausible explanation for the limited genetic exchange between strains from trees and pigeons because mopane and other native trees are found in rural areas, but pigeons inhabit urban centers.

### Multilocus analyses and population genetics indicate that global strains are highly clonal, and African strains are highly variable

Multilocus analysis confirmed the extraordinary genetic diversity of the native African isolates of *C. neoformans* var. *grubii* (Figures 1 and 2). The eight unlinked MLST loci identified 65 unique genotypes among the African isolates (35 VNI, 29 VNB and 1 VNII), but only 16 genotypes were detected in the global sample (12 VNI and 4 VNII). Among strains with the VNI molecular type, 26 genotypes were found only in southern Africa, nine genotypes were found on the five major continents, including Africa, and three genotypes were detected in the global sample but not in Africa (Fig. 1). The high genetic diversity of the African populations was confirmed by analyses of individual loci. For example, 11 non-recombinant haplotypes of *GPD1* were detected in Africa, but only three *GPD1* haplotypes were found among strains isolated from the rest of the world (Table 1). Similarly, six *URA5* haplotypes were found in the African sample, but there were only three in the global population. Overall, the eight loci revealed African to global haplotype ratios ranging from 2∶1 for *URA5* to 14∶1 for *SOD1* (Table 1).

**Table 1:** Numbers of haplotypes and predicted recombinational events at each locus in the African and Global populations of *C. neoformans* var. *grubii*, estimated by RECMIN and ARG [18], [19].

Locus	Population^a^	Total no. of haplotypes	No. of non-recombinant haplotypes	Minimal no. of recombinational events
*GPD1*	African	12	11	1
	Global	3	3	
*URA5*	African	12	6	2
	Global	3	3	
*SOD1*	African	15	14	6
	Global	1	1	
*TEF1*	African	16	16	0
	Global	3	3	
*CAP59*	African	7	6	1
	Global	2	2	
IGS1	African	14	13	2
	Global	4	2	
*PLB1*	African	10	9	1
	Global	3	3	
*LAC1*	African	9	7	4
	Global	4	3	

a The “African” population includes environmental and clinical isolates from Botswana and South Africa; the “Global” population includes environmental and clinical isolates from other locations. All 142 isolates are listed in Table S2.

The high genetic variability in the African population may be attributable to genetic exchange, the ancestral origin of the African population, or both. To determine the contribution of recombination and mutations to the genetic composition of the haplotypes, site compatibility matrices for each locus were generated, and putative recombinational events were identified by using RECMIN software [18], which calculates the minimal number of recombinational events in the history of the sample. RECMIN detected no evidence of recombination in the *TEF1* locus, but identified from one to six recombinational events in the other loci (Table 1). In addition, the extent of recombination in the phylogenetic history of each locus was evaluated by inferring minimal ancestral recombination graphs (ARGs) using the BEAGLE software [19] implemented in a SNAP Workbench [20], [21]. These networks, which represent the most parsimonious reconstructions of haplotype evolution with the assumption of recombination, support the results obtained by RECMIN (Fig. S3).

To confirm that the indigenous African population exhibited the highest genetic diversity, we compared a sample of 99 Botswanan and South African clinical and arboreal isolates with a set of 57 previously genotyped global strains that included every available, unique genotype [10]. For each of these samples, recombinant haplotypes were excluded, and the standard diversity indices for each population were calculated. As indicated in Table 2, the indigenous African population exhibited the highest genetic diversity. Gene diversity (*h*) is the probability that two randomly compared haplotypes in the sample are different [22], nucleotide diversity (*π*) is the probability that two random homologous nucleotides are different ** [23], and the.pairwise difference (*d*) is the mean number of base-pair differences between all pairs of haplotypes in the sample. With the exception of the highly homogeneous *CAP59* locus, *h*, *π* and *d* indices were higher among the African tree isolates compared to the global population sample (Table 2).

**Table 2:** African isolates of *C. neoformans* var. *grubii* are more diverse than the global population: comparison of the diversity indices and neutrality tests at each locus.

Locus	Population^a^	No. of isolates^b^	Gene diversity	Nucleotide diversity	Mean pairwise difference	Tajima's
			(*h*)	(*π*)	(*d*)	*T_D_* c
*GPD1*	African	99	0.86	0.005	2.3	−0.67
	Global	57	0.35	0.001	0.51	**−1.8**
*TEF1*	African	99	0.84	0.003	2.01	1.07
	Global	57	0.72	0.002	1.29	**−1.62**
*CAP59*	African	97	0.6	0.002	0.97	**−0.38**
	Global	47	0.61	0.002	1.01	−1.6
*PLB1*	African	80	0.77	0.003	1.89	−1.12
	Global	57	0.68	0.002	0.94	−0.89
IGS1	African	93	0.81	0.01	7.02	**−1.7**
	Global	41	0.23	0.0008	0.57	**−1.7**
*SOD1*	African	94	0.6	0.009	5.17	0.4
	Global	57	0.03	0.0005	0.28	−2.1
*LAC1*	African	88	0.82	0.006	2.67	0.45
	Global	49	0.73	0.004	1.3	1.02
*URA5*	African	92	0.75	0.007	4.7	0.24
	Global	47	0.6	0.001	1	**−1.59**

a The diversity of clinical and environmental strains of *C. neoformans var. grubii* from Botswana and South Africa was compared with that of a selection of the most genetically diverse global isolates available [10]. The “African” population included all clinical and avian isolates of *C. neoformans var. grubii* (85 strains) as well as 14 isolates from African trees; that is, we analyzed only one isolate per tree because multiple isolates from the same tree and surrounding soil were clonal. The “Global” sample was comprised of a larger sample of 57 previously genotyped, non-arboreal strains from 14 different countries (excluding Botswana and South Africa), and they included multiple isolates of each unique MLST or AFLP genotype [10]. Except for the *CAP59* locus, this purposely enlarged and intentionally diversified global sample revealed less variation (i.e., lower *h*, *π* and *d* values) than the sample of African isolates.

b We excluded strains with recombinant haplotypes at a locus.

c Values of Tajima's *T_D_* for which the null hypothesis of neutrality was rejected are bolded.

### Phylogenetic analysis indicates that the ancestral haplotypes of individual MLST loci are found in the African population of *C. neoformans* var. *grubii*

**Figure 3:**
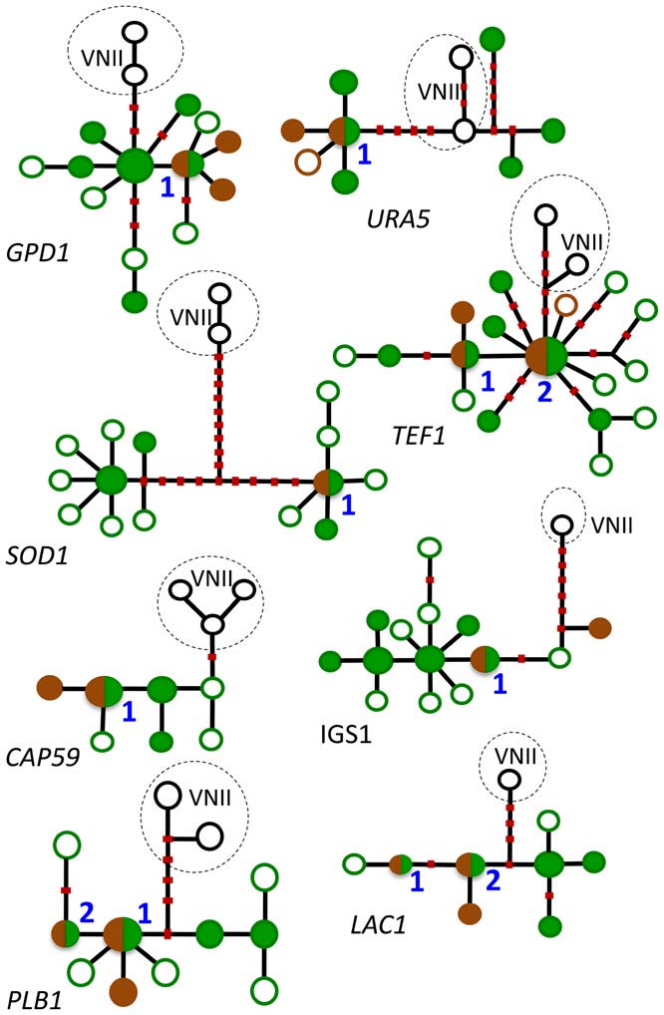
Haplotype networks of the eight MLST loci. Haplotypes of strains of *C. neoformans* var. *grubii* that have never been found outside Africa are shown in green: filled green circles designate haplotypes of strains that were obtained from trees (most were also found in patients), and empty green circles signify haplotypes that were obtained only from patients. Cosmopolitan haplotypes are shown in brown: filled brown circles designate haplotypes of strains from pigeon excreta (most were also found in patients), and empty brown circles signify haplotypes that were obtained only from patients. Circles that are half green and half brown, designated “1” and “2”, indicate haplotypes of strains found in trees and pigeon excreta, and they represent the ancestral haplotypes of global strains. Haplotypes from the global VNII subpopulation of *C. neoformans* var. *grubii* are used as an outgroup; they are shown in black and lightly encircled. Ancestral haplotypes are internal, and derived haplotypes occupy apical positions. Red dots on the lines connecting the haplotypes represent the most parsimonious number of mutational steps required to generate the allelic polymorphisms. Recombinant haplotypes identified by ARGs are excluded. The number of haplotypes per locus can also be observed. For example, 11 *GPD1* haplotypes were detected in Africa (empty, solid or half-filled green circles), and only three *GPD1* haplotypes were found among the global strains (empty, solid or half-filled brown circles). (One exception is the *TEF1* locus, which has 16 African haplotypes, but only 15 are depicted because one haplotype was found in a pigeon strain [D16-16].) Overall, the non-recombinant African to global haplotype ratios varied from 6 to 3 for *URA5* to 14 to 1 for *SOD1* (Table 1).

Features of the African strains of *C. neoformans* var. *grubii* resemble the putative ancestral population, such as (i) high genetic diversity, (ii) strong association with the geographic region, and (iii) a unique ecologic niche in native African trees. In contrast, the global population exhibits signs of recent expansion and/or bottleneck, such as (i) low genetic diversity and high clonality, (ii) association with the non-native, ubiquitous avian ecological niche, and (iii) lack of geographic structure. To test these properties, we reconstructed haplotype networks of each MLST locus [24]. This analysis utilizes statistical parsimony to infer phylogenetic relationships among haplotypes (Fig. 3). That is, internal nodes represent ancestral haplotypes from which the derived (distal) haplotypes evolved. Numerous haplotypes from the endemic African population of *C. neoformans* var. *grubii* occupy both internal (ancestral) and apical (derived) positions on the networks (Fig. 3, green circles). Conversely, haplotypes that are unique to the global population are scarce, almost always occupy apical positions in the networks, which suggest a more recent origin, and are always associated with strains from pigeon habitats (Fig. 3, brown circles). The ancestral haplotypes for all eight loci are found in isolates from African trees. Thus, the combined evidence of all the MLST loci suggests that (i) the ancestral population of *C. neoformans* var. *grubii* is associated with native African trees and (ii) the global population is a product of a range expansion of the ancestral African population.

As illustrated in Figure 3, the haplotype network of each locus identified putative ancestral haplotypes, from which all other global haplotypes can be derived. These haplotypes are centrally located on each haplotype network. They are present in both global strains, which are associated with pigeons, and endemic African strains, which are associated with trees. In Figure 3, these haplotypes are depicted as half green-half brown circles labeled “1” and “2.” For the *GPD1, URA5, SOD1, CAP59* and IGS1 loci, a single ancestral haplotype was detected, and for the *TEF1, PLB1* and *LAC1* loci, two ancestral haplotypes were observed. These results suggest that the emergence of only two strains possessing all eight ancestral haplotypes can explain the diversity among the global coprophilic population. Reading the haplotypes in Figure 3 from left to right, the ancestry of any extant global strain could have evolved from strains with “11111111” and “11121122” genotypes (or “11121111” and “11111122”, “11121121” and “11111112” or “11111121” and “11121112”).

**Figure 4:**
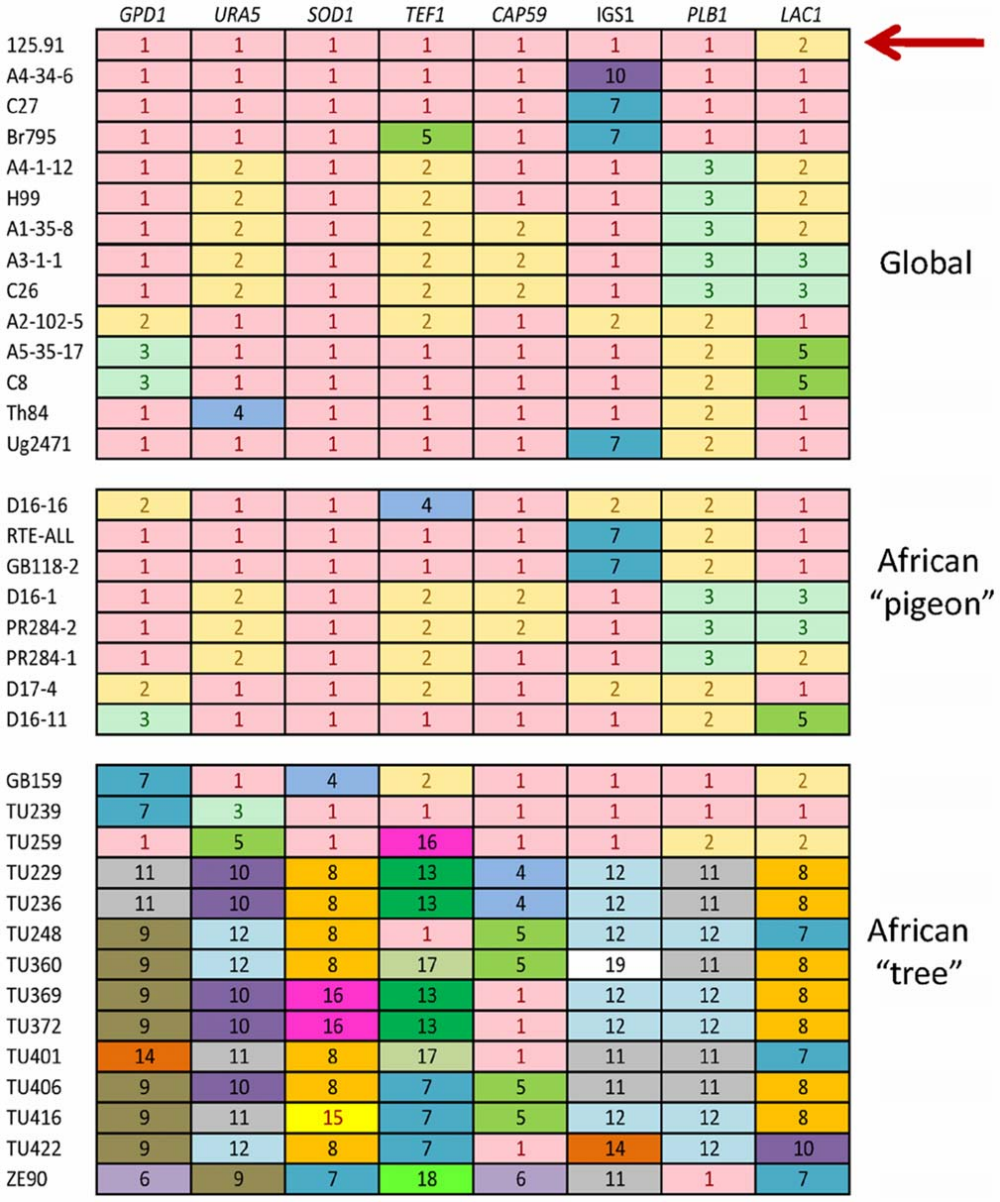
Comparison of representative allelic profiles of isolates of *C. neoformans* var. *grubii* from (top panel) the global population (VNI), including seven clinical and seven pigeon isolates, (middle panel) the coprophilic African population associated with pigeons (VNI), and (bottom panel) the African population associated with trees (VNB and VNI). Strain designations (see Table S2) are listed on the left. Under each locus, identical haplotypes are denoted with the same number and color. Haplotypes that are associated with both pigeons and trees are shown in pink (# 1) and yellow (# 2), respectively, and they represent ancestral haplotypes of the global population. The ancestral MLST genotype that might have emerged from Africa is marked with a red arrow. Emergence of any two strains comprised only of pink and yellow progenitor haplotypes can explain diversity in the contemporary global population of VNI.

Remarkably, we have identified strains that carry the ancestral haplotypes at all eight loci. As shown in Figure 4, a clinical isolate (strain 125.91, red arrow) has the “11111112” genotype, but it also has the rare *MAT* **a** mating type allele, which does not make it a likely candidate for a global ancestor because the *MAT* **a** allele is exceedingly rare among global isolates. (Strain 125.91 was the first isolate reported to possess the *MAT* **a** mating type [25], and it was subsequently used to generate a pair of congenic strains of *C. neoformans* var. *grubii* [14].) Several other clinical and environmental strains in the global population, such as strains A4-34-6 and C27 (Figure 4A), possess 7 of the 8 ancestral global haplotypes and the common *MATα* allele (Table S2).

### Population genetic analyses support the model of recent global expansion of the African population

Under the model of rapid population expansion, gene (*h*) and nucleotide (*π*) diversities and the mean pairwise sequence differences between the haplotypes (*d*) are expected to be low [26], [27]. As shown in Table 2, the data support these expectations. Compared with the African arboreal strains, the values of *h*, π and *d* for seven loci were lower in the global population sample.

Tests for statistical neutrality can infer the demographic history of a population [28]. Results of the neutrality test support the hypothesis of global expansion of *C. neoformans* var. *grubii*, as significantly negative *T_D_* values were obtained for 5 loci in the global population sample, but not in the native African arboreal population (Table 2). Negative *T_D_* values may be a consequence of selective sweep or expansion of the population size. However, the most likely explanation for significantly negative values of *T_D_* at half the loci is population expansion [27].

### Evidence for recombination among global and African populations of *C. neoformans* var. *grubii*

Strains that retain most of the ancestral haplotypes, such as A4-34-6 or C27 (Fig. 4), are prevalent in the global population. Each of these strains possesses seven ancestral global haplotypes and an unusual IGS1 haplotype, which is not found in African arboreal isolates. These atypical IGS1 alleles have considerable sequence similarity to IGS1 alleles in the VNII subpopulation, which suggests that they might have been acquired by recombination between strains of the VNI and VNII clades (Fig. S3). Our previous data and that of others indicate that the global population of *C. neoformans* var. *grubii* is predominantly clonal [8], [10], [29]; however, global samples exhibit limited evidence of recombination [11], [30], [31], which may have resulted from the recently discovered phenomenon of same sex mating in *C. neoformans* [12], [32]. The presence of an unusual IGS1 allele in the otherwise ancestral genotype of strain A4-34-6 (and other strains with the same genotype) supports the occurrence of occasional recombination between global strains (Fig. S3).

**Figure 5:**
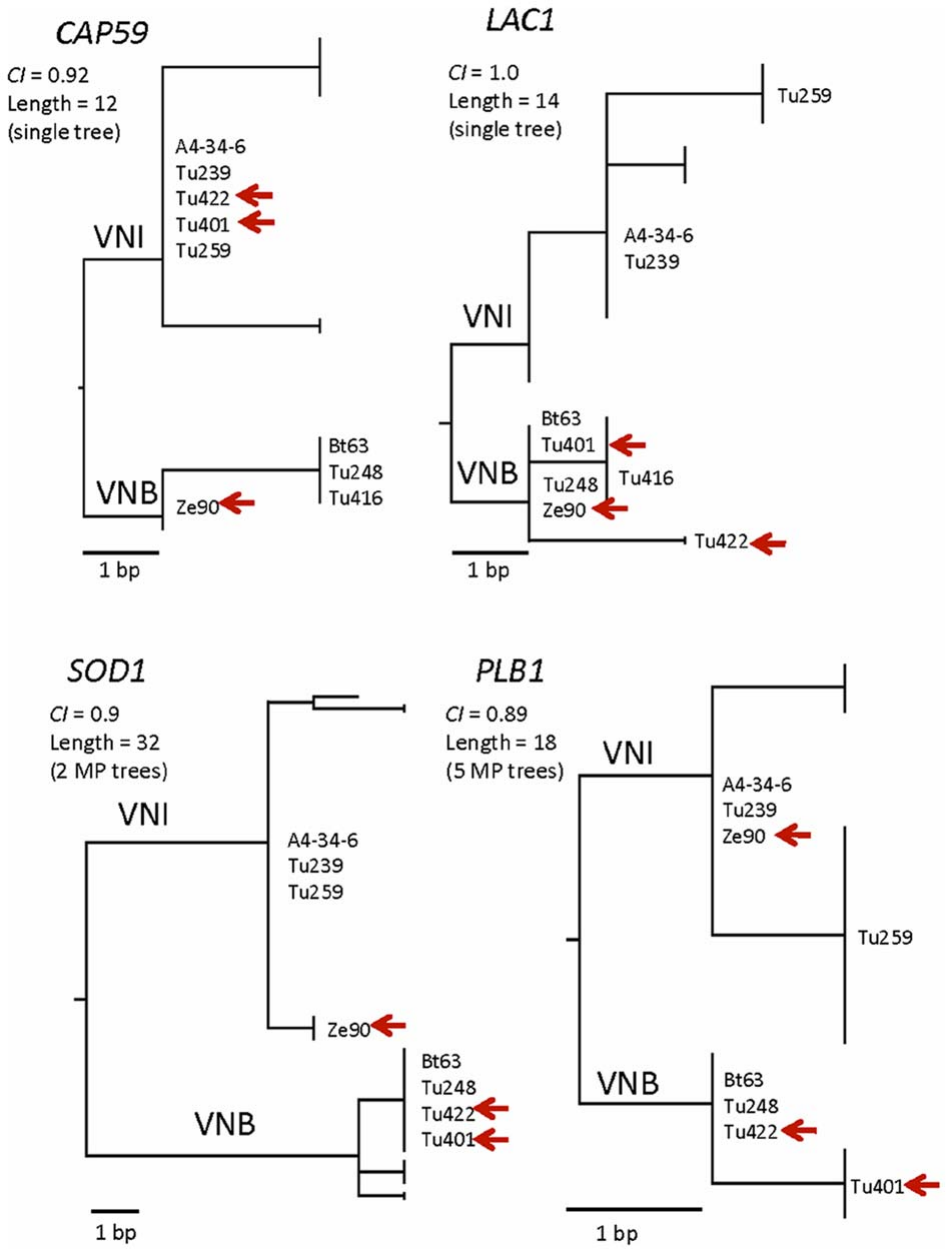
Incongruence among four gene genealogies of *C. neoformans* var. *grubii* obtained by maximum parsimony. For clarity, only representative stains are shown. The VNI and VNB subpopulations are indicated. Isolates that are inconsistently placed within the gene genealogies are marked with arrows.

Compared with the global population sample, linkage disequilibrium among the haplotypes is significantly lower in the native African population sample, which suggests a much higher level of genetic recombination among African strains. Previously, we reported evidence of recombination in a clinical sample of *C. neoformans* var. *grubii* isolates from Botswana [13]. Several lines of evidence here support recombination in the environmental population in Africa. (i) Isolates with the rare *MAT* **a** allele have been isolated from mopane tree bark (Table S2). (ii) Linkage equilibrium in arboreal African isolates was detected by measuring the standardized index of association (*I_A_*) in the population [33] (Table S3). (iii) Visual inspection of individual gene genealogies indicates obvious incongruence among the phylogenies of several loci. In Figure 5, compare the placement of strains Tu422, Tu401 and Ze90 on different branches of the individual phylograms. (iv) Recombination in African *C. neoformans* var. *grubii* strains is also supported by results of the Templeton and Kishino-Hasegawa tests [34], [35], [36], which detected significant incongruence, respectively, in 54 (96%) and 46 (82%) of 56 reciprocal pairwise comparisons among the eight loci (Table S4, *p*<0.05). In contrast, both tests support congruence among the gene genealogies of seven loci in the global population (Table S4), reinforcing clonality, which could be due to inbreeding as well as mitosis.

### VNI and VNB subpopulations share a common phylogenetic history, indicating that neither group is a cryptic species

Previously, we demonstrated that global populations of *C. neoformans* var. *grubii* consist of three genetically isolated subpopulations, VNI, VNII, and VNB [10]. Data presented here support this observation and confirm genetic isolation among three subpopulations (Fig. 1). However, phylogenetic analyses of each of the 8 individual loci indicated a monophyletic origin and fixation of polymorphic sites in the VNII subpopulation, which is consistent with the hypothesis that VNII represents a cryptic species [37]. The phylogenies of three representative loci in Figure S4 show that polymorphic sites are not reciprocally fixed in the VNI and VNB subpopulations. As noted above, the Templeton and Kishino-Hasegawa tests detected incongruence among the gene genealogies of the 8 loci, which indicates recombination and/or incomplete lineage sorting between the VNI and VNB populations. This result is inconsistent with the concept of species recognition by genealogical concordance [38]. In addition, phylogenetic analysis indicates that the VNI and VNB subpopulations share ancestral haplotypes at three loci (*CAP59, TEF1*, and *PLB1*), which indicates that these subpopulations share a common origin (Fig. S4). The evidence of both phylogenetic and population genetic analyses demonstrate that the VNI and VNB groups are not sufficiently diverged into cryptic species. Consequently, the high genetic variability of the VNB strains supports the hypothesis that global strains of VNI as well as VNB originated in Africa.

## Discussion

This investigation determined that African strains of *C. neoformans* var. *grubii* are more diverse than the global population. We discovered endemic strains of VNB and VNI that are found only in Africa and associated with native African trees, especially Mopane trees. We also identified global strains of VNI that exist within and out of Africa, and they are typically associated with columbine habitats. However, the African VNI strains are more diverse than the global strains of VNI. Phylogenetic analysis indicated that VNI and VNB strains are closely related and share a common phylogenetic history. These results are consistent with the parsimonious conclusion that the global population of VNI originated in Africa. Nevertheless, the converse may be true; African VNI isolates may have originated elsewhere and become more diverse in Africa.

These two hypotheses are not mutually exclusive. The “into Africa” model presupposes that VNI strains evolved elsewhere and were introduced to southern Africa by European colonists who brought pigeons (i.e., rock doves) to Africa. These introduced strains could have mated with the native African VNB population, producing genetically diverse haploid populations of VNI and VNB. Mating between VNI and VNB strains occurs in laboratory, and mating in nature is supported by the phylogenetic data (Fig. 5). This model is also consistent with the natural history of rock doves (*Columba livia*), whose feces provide the predominant ecological niche for VNI strains. Columbine birds are native to the Mediterranean basin, but they were introduced to many parts of the world, including southern Africa, during the European expansion that began 500 years ago [39], [40].

The “out-of Africa” model for the evolution of VNI strains suggests that the ancestral population is endemic to southern Africa and may have an ecological niche in native trees, such as the mopane. This model hypothesizes that after the introduction of columbines to Africa, a small number of diverse African strains established a new ecological niche in their excreta, where they proliferated clonally, became isolated genetically, and were eventually transported throughout the world by early traders and international commerce. As noted above, the “out-of-Africa” model is supported by the higher genetic diversity among the African VNI and VNB strains. The high genetic diversity of the African population of VNI is also apparent in the haplotype networks. Figure 3 illustrates that when putative recombinant haplotypes are excluded, three loci (*SOD1, TEF1* and *CAP59*) revealed more unique VNI haplotypes (6, 4 and 5, respectively) than the global VNI strains. At the other five loci, the African and global VNI haplotypes are comparable, but no global haplotypes outnumber those from Africa. Thus, cumulative evidence to date supports the “out-of-Africa” model for the origin of *C. neoformans* var. *grubii* strains. Other hypotheses would include the possibility that an accelerated rate of mutation among the African strains gave rise to their diversity, but there is no evidence to support this theory. In contrast to African strains, the global VNI strains are significantly clonal, and only three genotypes were unique to the global population (Fig. 1). Furthermore, to date, no other sites of genetic diversity have been identified in the global population of VNI strains.

Like any inference in population genetics, this model is based on the assumption that the global and African populations of *C. neoformans* var. *grubii* have been adequately sampled. For this investigation, we selected representative global strains after genotyping more than 1,000 strains of *C. neoformans* var. *grubii* from 15 countries [8], [10], [41], [42]. We included representatives of each MLST genotype found in each country. This sampling strategy allowed us to compile a comprehensive sample of the genetic diversity in the global population of *C. neoformans* var. *grubii*. Nevertheless, it is always possible that further sampling of clinical and environmental populations will uncover additional foci of genetic diversity. The proposed expansion of *C. neoformans* var. *grubii* from Africa may only pertain to the origin of strains associated with columbine birds, and additional research may reveal global strains of *C. neoformans* var. *grubii* that are associated with other ecological niches. For example, recently described *C. neoformans* var. *grubii* strains isolated from the decayed wood and soil in India may have a different origin [7], [43]. Similarly, the conclusions of this study are based on a relatively small sample of environmental isolates from southern Africa. Further environmental sampling may uncover additional ecological niches and/or centers of diversity in Africa.

The results here indicate that the genetic diversity among global *C. neoformans* var. *grubii* strains can be explained by the emergence of as few as two MLST genotypes from the ancestral population in Africa. However, other data suggest the possibility of multiple expansions from Africa. For example, we previously discovered that many diploid AD hybrid strains possessing the rare *MAT* **a** mating type allele of serotype A descended from the endemic population in sub-Saharan Africa [44]. The genetic background of the African AD ancestors differs from the ancestral global population of *C. neoformans* var. *grubii*, which implies that the AD strains might have emerged independently from Africa. Similarly, a small number of VNB strains was recently found in South America [29], [37], and this finding may indicate another independent emigration from southern Africa.

Data presented here have several public health implications. We described a novel ecological niche for *C. neoformans* in Africa, the mopane tree. Almost 30% of *C. mopane* (mopane trees) in the southern Africa are colonized by highly genetically diverse and potentially virulent strains *of C. neoformans* var. *grubii*, which may prove to be a source of human infections. Mopane trees are endemic to southern Africa, and they contribute to the economy and culture [45], [46], [47], [48]. Mopane timber is frequently used for firewood and construction. For example, the walls and roofs of traditional huts in the eastern Limpopo Province of South Africa are constructed almost entirely of debarked mopane poles [49]. Mopane bark, wood, leaves and seeds are also used extensively in traditional medicine [48], [49]. Mopane trees are also the sole substrate for the cultivation of edible mopane worms, which are caterpillars of the *Gonimbrasia belina* moth, and a culinary delicacy in Botswana, South Africa, Angola, Namibia and Zimbabwe [50]. The constellation of (i) substantial contamination of southern African mopane trees by *C. neoformans* var. *grubii*, (ii) the high number of HIV-infected individuals in this region, and (iii) the popularity of mopane wood in traditional construction, medicine and cuisine raises the possibility that people at risk for cryptococcosis may be frequently exposed to *C. neoformans* var. *grubii*. This unique situation may impact the public health and warrant investigation.

The discovery in southern Africa of highly diverse progenitor strains of *C. neoformans* var. *grubii* with the capacity for sexual as well as clonal reproduction evokes several other scientific and public health implications. (i) If strains of *C. neoformans* var. *grubii* with enhanced pathogenicity emerge in the future, they are likely to have originated in southern Africa. (ii) Most current research on the virulence, genetics and genomics of *C. neoformans* var. *grubii*, as well as the pathogenesis and treatment of cryptococcal disease, are focused on a few laboratory strains that possess global genotypes, which descended from African strains. To ensure that the results of these many and varied studies are applicable to all extant and future isolates, they should focus on the more diverse African strains with ancestral genotypes. (iii) Sub-Saharan Africa is the global hotbed of AIDS and cryptococcosis. HIV and *C. neoformans* var. *grubii* co-evolved and are most diverse in this region, where clinical data show that the incidence, severity and mortality of co-infection are the highest. The interaction between these pathogens warrants investigation. (iv) This report provides a strategy to elucidate the origins of other pathogenic fungi.

## Materials and Methods

### Environmental sampling

We sampled 440 locations in South Africa and Botswana, and recovered 273 isolates of *C. neoformans* from 22 different sites (Fig. S1 and Table S1). The samples included water, soil, avian and mammalian excreta, animal burrows, termite mounds, plant debris, and leaves, bark and decayed wood of native and introduced species of trees. Air samples were taken with an RCS-Plus air sampler (Biotest Hycon, Denville, NJ). Isolates of *C. neoformans* were only recovered from pigeon feces, soil, decayed wood and tree bark (Table S1).

Sterile BBL^TM^ culture swabs containing Amies medium (BD Diagnostics, Franklin Lakes, NJ) were used to swab trees. The following species of trees were sampled: *Acacia* sp. (n = 45, 0 positive), *Adansonia digitata* (Baobab, n = 3, 1 positive), *Boscia albitrunca* (Shepherd's tree, n = 15, 0 positive), *Colophospermum mopane* (Mopane, n = 31, 9 positive), *Erythrina lysistemon* (Coral tree, n = 3, 0 positive), *Eucalyptus* sp. (n = 32, 1 positive), *Euphorbia ingens* (n = 4, 0 positive), *Ficus abutifolia* (n = 3, 0 positive*), Sclerocarya birrea* (Marula, n = 5, 0 positive); in addition, one of 21 unidentified trees was positive for *C. neoformans* var. *grubii*. Positive cultures were obtained from areas protected from direct sun-light, such as tree hollows and under the bark. No isolates were obtained from leaves, fruit or plant debris. For each tree, a corresponding soil sample was obtained from the root zone within approximately 1 m from the base of the tree and 10 cm below the surface. Two soil samples associated with the mopane trees and one sample from under a eucalyptus tree were positive for *C. neoformans* var. *grubii*.

Samples of soil and feces of birds and mammals were collected in sterile plastic tubes. Five of 41 samples of pigeon excreta were positive for *C. neoformans* var. *grubii*. No positive cultures were obtained from the excreta of other birds or animals. In addition, 17 soil samples were collected from areas that were not associated with trees, and one sample containing soil contaminated with the excreta of an unknown bird species was positive for *C. neoformans* var. *grubii* (Table S1).

For primary isolation of *Cryptococcus* from environmental samples, we used Staib's agar [51] supplemented with 0.2 g/L chloramphenicol (Sigma-Aldrich, St. Louis, MO), 0.025 g/L gentamicin (EM Science, Gibbstown, NJ) and 0.1 g/L (0.1 g/10 mL 95% ethanol) biphenyl (Alfa Aesar, Ward Hill, MA) [8]. Culture swabs were directly spread on duplicate Staib plates. Samples of soil and droppings were resuspended in 10 ml of sterile water by vortexing, the sediment was allowed to settle for approximately 10 min, a 1∶10 dilution was prepared in sterile water, and 50 µL of each suspension was spread on a Staib plate. Inoculated plates were incubated at 35–37°C for 3–5 d. Brown yeast colonies were selected, grown in pure culture on Staib's agar plates without antibiotics, confirmed to be *C. neoformans* by standard morphological and physiological criteria, and maintained on yeast extract-peptone-dextrose (YPD) agar (Difco, Baltimore, MD) at 30°C. All environmental samples were processed within 1–3 d after collection.

### Strains used in study

As listed in Table S2, a total of 142 strains were selected for MLST analyses, including 58 environmental isolates that represented all 22 positive sites (1–5 isolates per site) and 59 clinical strains obtained from South African and Botswanan patients in hospitals that were located relatively close to the environmental sampling sites. For comparison with a global sample of *C. neoformans* var. *grubii*, we selected 25 strains from elsewhere in the world that represented different MLST genotypes from a previously analyzed global collection of more than 1,000 isolates [10].

### Growth on mopane bark and pigeon feces

Culture media containing 12.5% pigeon excreta as the sole source of nutrients was prepared as previously described [15]. Mopane bark medium was prepared by boiling 100 g of mopane bark in 1 L sterile water for 30 min and filtering. The volume of the filtrate was adjusted to 1 L, 20 g agar was added, and the mixture was autoclaved for 20 min. Four strains of *C. neoformans* var. *grubii* isolated from pigeon feces, eight strains isolated from trees and two strains from the global sample were grown overnight in YPD, harvested, washed with sterile water, enumerated in a hemocytometer chamber, and adjusted to 1×10^8^ colony-forming units/mL. Serial ten-fold dilutions were prepared, and 2.0 µL of each strain was spotted onto plates of pigeon feces and mopane bark media. The plates were incubated at 37°C for 3 d, and the growth of each strain was assessed visually and compared.

### DNA manipulations and MLST

Genomic DNA was obtained using MasterPure^TM^ Yeast DNA purification kit (Epicentre Biotechnologies, Madison, WI). Eight previously described MLST loci were used to analyze genetic diversity of the sample: *CAP59*, *GPD1*, *IGS1*, *LAC1*, *PLB1*, *SOD1*, *URA5* and *TEF1* [10], [16], which included seven consensus MLST loci [16]. The PCR primers and amplification conditions are shown in Table S5. Each PCR mixture contained 20 µl of 1X PCR buffer, 2 mM MgCl_2_, 0.2 mM dNTPs, 1 µM each primer, 0.065 µL i*Taq* DNA Polymerase (Bio-Rad, Hercules, CA), and approximately 1 ng genomic DNA. PCR products were purified using ExoSap-IT purification method (Affymetrix, Cleveland OH), and sequenced using an ABI 3730xl sequencer with Big Dye terminators (Applied Biosystems). DNA sequencing reactions and PCR conditions for these loci were the same as previously described [10]. For all loci, PCR primers used to amplify the fragments were also used for sequencing. Sequences were generated from both DNA strands and edited manually.

All 142 strains were analyzed by MLST. Sequences were automatically aligned using Sequencher 4.1 (Gene Code Corporation); the alignment was imported into MacClade 4.05 [52] and edited manually. Ambiguously aligned characters and gaps were excluded from the analysis. MLST alleles were assigned to every unique sequence type at each locus, and an eight-digit number designated the allelic profile of each isolate.

### Determination of mating type

The mating type of each strain was identified by PCR using mating type- and serotype-specific primers that amplify portions of the *STE20* **a** or *STE20α* genes [25]. Results were confirmed by crossing with the *MATα* and *MAT* **a** reference strains (H99 and Bt63, respectively) on V8 juice agar as described [14].

### Assessing population structure

Using the Community Analysis Package 2.4 (PISCES Conservation Ltd., Hampshire, UK) with the correlation matrix, the genetic relatedness of MLST genotypes was evaluated by principal component analysis (PCA). The neighbor joining (NJ) method with uncorrected (“p”) genetic distances was used to analyze combined sequence data for all 142 isolates. The analysis was performed using PAUP version 4.0b10 [53] and visualized using Geneious Pro 5.1 [54]. The Arlequin 2.0 program [17], [55] was used to calculate the pairwise Wright's fixation indices (*F_ST_*) for the pairs of populations. The STRUCTURE 2.2 software was executed in the admixture with linkage disequilibrium model [56], [57] to assign strains to subpopulations (VNI, VNII and VNB).

### Phylogenetic analyses and tests for congruency

Maximum parsimony (MP) trees for the individual loci were identified with heuristic searches based on 500 random sequence additions for each data set. PAUP [58] was used to obtain maximum likelihood (ML) trees. For each locus, best-fit models of evolution were identified using hierarchical likelihood ratio test implemented in MODELTEST [59]. K80 model was used for *PLB1* and *CAP59* loci, and HKY model for *TEF1* locus. Haplotype networks for each locus were constructed using program TCS version 1.13 [60]. Incongruence among the MP trees was determined using Templeton and Kishino-Hasegawa tests implemented in PAUP.

### Assessing recombination

The multilocus 1.2 software [61] was used to calculate the standardized index of association (*I_A_*) and evaluate linkage disequilibrium among the loci. Because clonal reproduction is common among *C. neoformans* var. *grubii* isolates, clone-corrected samples were used for this analysis [33]. To distinguish between haplotypes that originated from recombination and those that arose by mutations, the RECMIN program [18] implemented in SNAP Workbench [20] was used to calculate site compatibility matrices of each locus, determine the recombination boundaries, and identify putative recombinant haplotypes. By reconstructing a minimal ancestral recombination graph (ARG) using the Branch and Bound algorithm of the BEAGLE software [19] implemented in SNAP Workbench [20], [21], we determined the relative order of recombinational events. The ARG assumes recombination and represents the most parsimonious reconstruction of the haplotype evolution. ARGs were rooted with the outgroup sequences of a strain of serotype D (JEC21), which is considered a sibling group of serotype A (*C. neoformans* var. *grubii*) [10], [31].

### Assessing molecular diversity

For each locus, putative recombinant haplotypes were excluded and standard diversity indices were calculated, including (i) gene diversity (*h*), the probability that any two random haplotypes in the sample are different [22], (ii) nucleotide diversity (*π*), the probability that any two random, homologous nucleotides are different [23], (iii) pairwise difference (*d*), the mean number of base pair differences between all pairs of haplotypes in the sample, and (iv) Tajima's *T_D_*, which tests the null hypothesis of equilibrium or selective neutrality in the evolutionary process. To challenge the hypothesis that the native African tree isolates are more genetically diverse than global isolates, the global population sample was selected to include the most diverse global strains available from our previous studies (Table 2). We included 57 strains from 14 different countries (excluding Botswana and South Africa); this sample included at least two isolates per country and every available clinical or environmental (pigeon) MLST/AFLP genotype (10). Conversely, the most genetically homogeneous sample of the African population was selected, which included: (i) clone-corrected, non-recombinant isolates from native Botswanan and South African trees, (ii) all non-recombinant isolates from pigeon habitats from Botswana and South Africa, and (iii) all non-recombinant isolates from patients admitted with cryptococcal meningitis to Botswanan or South African hospitals.

### DNA Accession Numbers

DNA sequences from this investigation were deposited in EMBL data base with the following accession numbers: *CAP59,* FN822780–FN822918; *GPD1*, FN826909–FN827047; IGS1, FN824659–FN824797; *PLB1*, FN824976–FN825114; *LAC1*, FN825115–FN825253; *SOD1*, FN825255–FN825393; *TEF1*, FN825394–FN825532; and *URA5*, FN825533–FN825537.

## Supporting Information

Figure S1 General geographical regions of Botswana (BW) and the Republic of South Africa (RSA) where environmental (red triangles) and clinical (blue triangles) isolates were obtained.
(PDF)

Figure S2 The growth of African arboreal and avian strains on mopane bark and pigeon excreta media. *C. neoformans* var. *grubii* are able to grow in the laboratory on media containing 10% boiled mopane bark (left) or 12% pigeon excreta (right) as sole nutrients. All strains except the Botswanan isolate of *Cryptococcus gattii* (a sibling species of *C. neoformans* var. *grubii* obtained from an unidentified tree) are listed in Table S2. D17-1, D16-1, Gb118-1, and Jo278-1 are strains of VNI (mating type α) that were isolated from samples of pigeon feces in the Republic of South Africa (RSA) or Botswana (BW); H99 (VNI, α) and Bt63 (VNB, **a**) were isolated from patients in the USA and BW, respectively; Tu406-1 (VNB, α), Tu422-1 (VNB, **a**), Tu259-1 (VNI, α), Tu-241-1 (VNI, α) and Tu372-1 (VNB, α) were isolated from mopane trees in BW; Gb159-1 (VNI, α) was isolated from an unidentified tree in BW; and Ze90-1 (VNB, α) was isolated from a *Eucalyptus* tree in RSA. Yeast cells were grown overnight in yeast nitrogen broth, washed, enumerated, and 10-fold serial dilutions were plated and incubated at 37°C for 48 hours.
(PDF)

Figure S3 Ancestral recombination graphs (ARGs) of the eight MLST loci. Each ARG is rooted with serotype D sequence (H1). Blue ellipses designate the recombination nodes, and the numbers inside them indicate the SNP immediately to the left of the recombination breakpoint. The paths leading to the recombination nodes are labeled with a P (prefix) or S (suffix), indicating the 5′ and 3′ segments of the recombinant sequence, respectively. Numbers next to the branches signify the number of mutational steps between the haplotypes; the absence of a number indicates that the haplotype did not change. Ecological and geographic origins of the haplotypes are mapped on the ARGs: Green ellipses indicate an ecological niche in trees, and brown ellipses designate an ecological niche in pigeon feces. Empty green ellipses denote clinical strains endemic to Africa, and empty brown ellipses represent global clinical strains.
(PDF)

Figure S4 Genealogies of *CAP59*, *PLB1* and *TEF1* loci obtained using maximum likelihood method. Strains are color-coded based on their assignment to different subpopulations: VNII strains are green, VNB strains are red, VNI strains are blue. Strains are assigned to subpopulations based on NJ analysis of the concatenated loci (Fig. 2) and Bayesian algorithm implemented in software Structure. Numbers show >60% bootstrap support for clades. Clades that include both VNI and VNB strains are bolded. For clarity of presentation the number of strains is reduced (approximately 30% strains with identical genotypes were removed for clarity). Gene genealogies are unrooted.
(PDF)

Table S1 Environmental samples yielding isolates of *Cryptococcus neoformans* var. *grubii.*
(PDF)

Table S2 Descriptions of the 142 strains of *Cryptococcus neoformans* var. *grubii* used in this study.
(PDF)

Table S3 Indices of association (*I_A_*) among the loci in subpopulations of *C. neoformans* var. *grubii*.
(PDF)

Table S4 Results of Templeton (T) and Kishino-Hasegawa (K-H) tests for conflict among phylogenetic topologies of the gene genealogies at each locus.
(PDF)

Table S5 MLST primers and PCR conditions used in this study.
(PDF)

## References

[1] Idnurm A, Bahn YS, Nielsen K, Lin X, Fraser JA (2005). Deciphering the model pathogenic fungus *Cryptococcus neoformans*.. Nat Rev Microbiol.

[2] Chayakulkeeree M, Perfect JR (2006). Cryptococcosis.. Infect Dis Clin North Am 20: 507-544, v-.

[3] Chuck SL, Sande MA (1989). Infections with *Cryptococcus neoformans* in the acquired immunodeficiency syndrome.. N Engl J Med.

[4] Park BJ, Wannemuehler KA, Marston BJ, Govender N, Pappas PG (2009). Estimation of the current global burden of cryptococcal meningitis among persons living with HIV/AIDS.. AIDS.

[5] Dromer F, Mathoulin-Pelissier S, Launay O, Lortholary O (2007). Determinants of Disease Presentation and Outcome during Cryptococcosis: the CryptoA/D Study.. PLoS Med.

[6] Mirza SA, Phelan M, Rimland D, Graviss E, Hamill R (2003). The changing epidemiology of cryptococcosis: an update from population-based active surveillance in 2 large metropolitan areas, 1992-2000.. Clin Infect Dis.

[7] Hiremath SS, Chowdhary A, Kowshik T, Randhawa HS, Sun S (2008). Long-distance dispersal and recombination in environmental populations of *Cryptococcus neoformans* var. *grubii* from India.. Microbiology.

[8] Litvintseva AP, Kestenbaum L, Vilgalys R, Mitchell TG (2005). Comparative analysis of environmental and clinical populations of *Cryptococcus neoformans*.. J Clin Microbiol.

[9] Littman ML, Borok R (1968). Relation of the pigeon to cryptococcosis: natural carrier state, heat resistance and survival of *Cryptococcus neoformans*.. Mycopathologia et Mycologia Applicata.

[10] Litvintseva AP, Thakur R, Vilgalys R, Mitchell TG (2006). Multilocus sequence typing reveals three genetic subpopulations of *Cryptococcus neoformans* var. *grubii* (serotype A), including a unique population in Botswana.. Genetics.

[11] Nielsen K, Heitman J (2007). Sex and virulence of human pathogenic fungi.. Adv Genet.

[12] Lin X, Hull CM, Heitman J (2005). Sexual reproduction between partners of the same mating type in *Cryptococcus neoformans*.. Nature.

[13] Litvintseva AP, Marra RE, Nielsen K, Heitman J, Vilgalys R (2003). Evidence of sexual recombination among *Cryptococcus neoformans* serotype A isolates in sub-Saharan Africa.. Eukaryot Cell.

[14] Nielsen K, Cox G, Wang P, Toffaletti D, Perfect JR (2003). Sexual cycle of *Cryptococcus neoformans* var. *grubii* and virulence of congenic **a** and α isolates.. Infect Immun.

[15] Nielsen K, De Obaldia AL, Heitman J (2007). *Cryptococcus neoformans* mates on pigeon guano: implications for the realized ecological niche and globalization.. Eukaryot Cell.

[16] Meyer W, Aanensen DM, Boekhout T, Cogliati M, Diaz MR (2009). Consensus multi-locus sequence typing scheme for *Cryptococcus neoformans* and *Cryptococcus gattii*.. Med Mycol.

[17] Wright S (1978). Variability within and among Natural Populations.. Evolution and the Genetics of Populations.

[18] Myers SR, Griffiths RC (2003). Bounds on the minimum number of recombination events in a sample history.. Genetics.

[19] Lyngso RB, Song YS, Hein J (2008). Accurate Computation of likelihoods in the coalescent with recombination via parsimony.. Lect Notes Comput Sci.

[20] Price EW, Carbone I (2005). SNAP: workbench management tool for evolutionary population genetic analysis.. Bioinformatics.

[21] Aylor DL, Price EW, Carbone I (2006). SNAP: Combine and Map modules for multilocus population genetic analysis.. Bioinformatics.

[22] Nei M (1973). Analysis of gene diversity in subdivided populations.. Proc Natl Acad Sci U S A.

[23] Nei M, Li W-H (1979). Mathematical model for studying genetic variation in terms of restriction endonucleases.. Proc Natl Acad Sci U S A.

[24] Templeton AR (2005). Haplotype trees and modern human origins.. Am J Phys Anthropol.

[25] Lengeler KB, Wang P, Cox GM, Perfect JR, Heitman J (2000). Identification of the MAT**a** mating-type locus of *Cryptococcus neoformans* reveals a serotype A MAT**a** strain thought to have been extinct.. Proc Natl Acad Sci U S A.

[26] Excoffier L, Smouse PE, Quattro JM (1992). Analysis of molecular variance inferred from metric distances among DNA haplotypes: application to human mitochondrial DNA restriction data.. Genetics.

[27] Eswaran V, Harpending H, Rogers AR (2005). Genomics refutes an exclusively African origin of humans.. J Hum Evol.

[28] Fu YX, Li WH (1993). Statistical tests of neutrality of mutations.. Genetics.

[29] Bovers M, Hagen F, Kuramae EE, Boekhout T (2008). Six monophyletic lineages identified within *Cryptococcus neoformans* and *Cryptococcus gattii* by multi-locus sequence typing.. Fungal Genet Biol.

[30] Bui T, Lin X, Malik R, Heitman J, Carter D (2008). Isolates of *Cryptococcus neoformans* from infected animals reveal genetic exchange in unisexual, alpha mating type populations.. Eukaryot Cell.

[31] Xu J, Vilgalys R, Mitchell TG (2000). Multiple gene genealogies reveal recent dispersion and hybridization in the human pathogenic fungus *Cryptococcus neoformans*.. Mol Ecol.

[32] Fraser JA, Giles SS, Wenink EC, Geunes-Boyer SG, Wright JR (2005). Same-sex mating and the origin of the Vancouver Island *Cryptococcus gattii* outbreak.. Nature.

[33] Smith JM, Smith NH, O'Rourke M, Spratt BG (1993). How clonal are bacteria?. Proc Natl Acad Sci U S A.

[34] Kishino H, Hasegawa M (1989). Evaluation of the maximum likelihood estimate of the evolutionary tree topologies from DNA sequence data, and the branching order in hominoidea.. J Mol Evol.

[35] Templeton AR (1983). Phylogenetic inference from restriction endonuclease cleavage site maps with particular reference to humans and apes.. Evolution.

[36] Felsenstein J (2004). Inferring Phylogenies..

[37] Ngamskulrungroj P, Gilgado F, Faganello J, Litvintseva AP, Leal AL (2009). Genetic diversity of the *Cryptococcus* species complex suggests that *Cryptococcus gattii* deserves to have varieties.. PLoS ONE.

[38] Taylor JW, Jacobson DJ, Kroken S, Kasuga T, Geiser DM (2000). Phylogenetic species recognition and species concepts in fungi.. Fungal Genet Biol.

[39] Mooney HA, Hobbs RJ (2000). Invasive species in a changing world..

[40] Grzimek B, Schlager N, Olendorf D,  McDade MC (2004). Grzimek's animal life encyclopedia;. Detroit Gale.

[41] Chen J, Varma A, Diaz MR, Litvintseva AP, Wollenberg KK (2008). *Cryptococcus neoformans* strains and infection in apparently immunocompetent patients, China.. Emerg Infect Dis.

[42] Choi YH, Ngamskulrungroj P, Varma A, Sionov E, Hwang SM (2010). Prevalence of the VNIc genotype of *Cryptococcus neoformans* in non-HIV-associated cryptococcosis in the Republic of Korea.. FEMS Yeast Res.

[43] Randhawa HS, Kowshik T, Chowdhary A, Preeti Sinha K, Khan ZU (2008). The expanding host tree species spectrum of *Cryptococcus gattii* and *Cryptococcus neoformans* and their isolations from surrounding soil in India.. Med Mycol.

[44] Litvintseva AP, Lin X, Templeton I, Heitman J, Mitchell TG (2007). Many globally isolated AD hybrid strains of *Cryptococcus neoformans* originated in Africa.. PLoS Pathog.

[45] Hempson GP, February EC, Verboom GA (2007). Determinants of savanna vegetation structure: Insights from *Colophospermum mopane*.. Austral Ecology.

[46] Sebego RJ, Arnberg W, Lunden B, Ringrose S (2008). Mapping of *Colophospermurn mopane* using Landsat TM in eastern Botswana.. South African Geographical Journal.

[47] Mapaure I (1994). The distribution of *Colophospermum mopane* (*Leguminosae-Caesalpinioideae*) in Africa.. Kirkia.

[48] Venter F, Venter J-A (1996). Making the most of indigenous trees..

[49] Mashabane LGW DCJ, Potgieter MJ (2001). The utilisation of *Colophospermum mopane* by the Vatsonga in the Gazankulu region (eastern Northern Province, South Africa).. South African Journal of Botany.

[50] Vogel G For moreprotein, filet of cricket (2010). Science.

[51] Staib F, Seibold M, Antweiler E, Frohlich B (1989). Staib agar supplemented with triple antibiotic combination for the detection of *Cryptococcus neoformans* in clinical specimens.. Mycoses.

[52] Maddison WP, Maddison DR (1989). Interactive analysis of phylogeny and character evolution using the computer program MacClade.. Folia Primatol (Basel).

[53] Swofford DL, Hill D, Moritz C, Mable B (1996). Phylogenetic inference.. Molecular systematics.

[54] Drummond AJ, Ashton B, Buxton S, Cheung M, Cooper A (2010). Geneios Pro V. 5.1.. http://www.geneious.com.

[55] Hartl DL, Clark AG (1997). Principles of Population Genetics..

[56] Pritchard JK, Stephens M, Donnelly P (2000). Inference of population structure using multilocus genotype data.. Genetics.

[57] Falush D, Stephens M, Pritchard JK (2003). Inference of population structure using multilocus genotype data: linked loci and correlated allele frequencies.. Genetics.

[58] Swofford DL (2002). PAUP*: phylogenetic analysis using parsimony (and other methods), version 4.0b..

[59] Posada D, Crandall KA (1998). MODELTEST: testing the model of DNA substitution.. Bioinformatics.

[60] Clement M, Posada D, Crandall KA (2000). TCS: a computer program to estimate gene genealogies.. Mol Ecol.

[61] Agapow PM, Burt A (2001). Indices of multilocus linkage disequilibrium.. Mol Ec Notes.

[62] Perfect JR (2005). *Cryptococcus neoformans*: a sugar-coated killer with designer genes.. FEMS Immunol Med Microbiol.

