# Supplementary figures and images for: Evidence that the Human Pathogenic Fungus Cryptococcus neoformans var. grubii May Have Evolved in Africa
Source: PLoS One. 2011 May 11;6(5):e19688. doi: 10.1371/journal.pone.0019688 (PMC3092753; doi:10.1371/journal.pone.0019688)

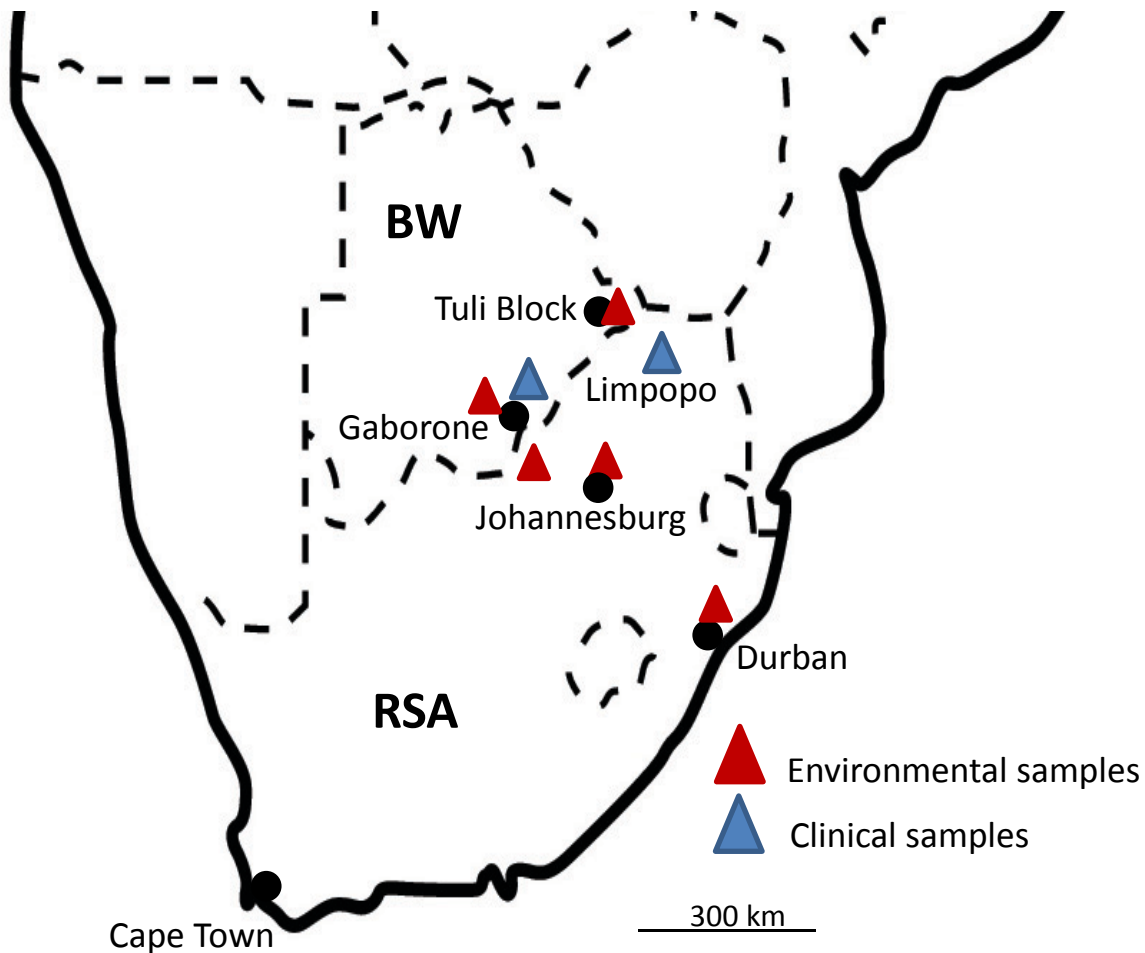

Supplement: Figure S1 — General geographical regions of Botswana (BW) and the Republic of South Africa (RSA) where environmental (red triangles) and clinical (blue triangles) isolates were obtained. (PDF) [file pone.0019688.s001.pdf]

Mopane bark

Pigeon Guano

D17-1

D16-1

Gb118-1

Jo278-1

H99

*C. gattii* (Tu294-1)

Bt63

Tu406-1

Tu422-1

Tu259-1

Gb159-1

Ze90-1

Tu241-1

Tu372-1

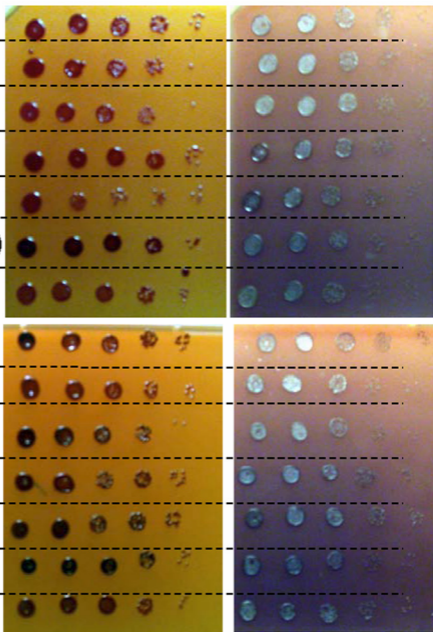

Supplement: Figure S2 — The growth of African arboreal and avian strains on mopane bark and pigeon excreta media. C. neoformans var. grubii are able to grow in the laboratory on media containing 10% boiled mopane bark (left) or 12% pigeon excreta (right) as sole nutrients. All strains except the Botswanan isolate of Cryptococcus gattii (a sibling species of C. neoformans var. grubii obtained from an unidentified tree) are listed in Table S2. D17-1, D16-1, Gb118-1, and Jo278-1 are strains of VNI (mating type α) that were isolated from samples of pigeon feces in the Republic of South Africa (RSA) or Botswana (BW); H99 (VNI, α) and Bt63 (VNB, a) were isolated from patients in the USA and BW, respectively; Tu406-1 (VNB, α), Tu422-1 (VNB, a), Tu259-1 (VNI, α), Tu-241-1 (VNI, α) and Tu372-1 (VNB, α) were isolated from mopane trees in BW; Gb159-1 (VNI, α) was isolated from an unidentified tree in BW; and Ze90-1 (VNB, α) was isolated from a Eucalyptus tree in RSA. Yeast cells were grown overnight in yeast nitrogen broth, washed, enumerated, and 10-fold serial dilutions were plated and incubated at 37°C for 48 hours. (PDF) [file pone.0019688.s002.pdf]

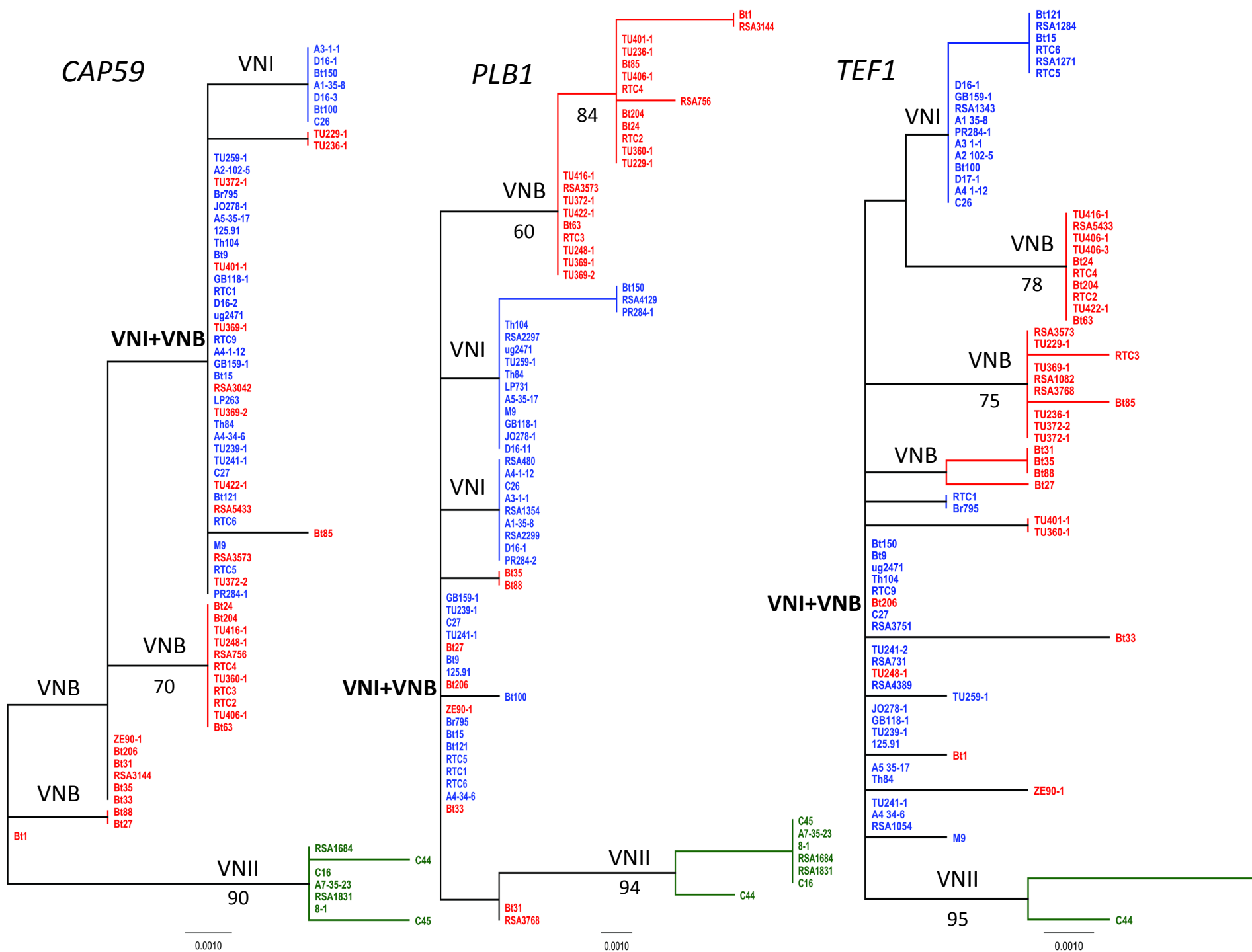

Supplement: Figure S4 — Genealogies of CAP59, PLB1 and TEF1 loci obtained using maximum likelihood method. Strains are color-coded based on their assignment to different subpopulations: VNII strains are green, VNB strains are red, VNI strains are blue. Strains are assigned to subpopulations based on NJ analysis of the concatenated loci (Fig. 2) and Bayesian algorithm implemented in software Structure. Numbers show >60% bootstrap support for clades. Clades that include both VNI and VNB strains are bolded. For clarity of presentation the number of strains is reduced (approximately 30% strains with identical genotypes were removed for clarity). Gene genealogies are unrooted. (PDF) [file pone.0019688.s004.pdf]
