# Supplementary material for: Evidence that the Human Pathogenic Fungus Cryptococcus neoformans var. grubii May Have Evolved in Africa
Source: PLoS One. 2011 May 11;6(5):e19688. doi: 10.1371/journal.pone.0019688 (PMC3092753; doi:10.1371/journal.pone.0019688)
Supplement: Figure S3 — Ancestral recombination graphs (ARGs) of the eight MLST loci. Each ARG is rooted with serotype D sequence (H1). Blue ellipses designate the recombination nodes, and the numbers inside them indicate the SNP immediately to the left of the recombination breakpoint. The paths leading to the recombination nodes are labeled with a P (prefix) or S (suffix), indicating the 5′ and 3′ segments of the recombinant sequence, respectively. Numbers next to the branches signify the number of mutational steps between the haplotypes; the absence of a number indicates that the haplotype did not change. Ecological and geographic origins of the haplotypes are mapped on the ARGs: Green ellipses indicate an ecological niche in trees, and brown ellipses designate an ecological niche in pigeon feces. Empty green ellipses denote clinical strains endemic to Africa, and empty brown ellipses represent global clinical strains. (PDF) [file pone.0019688.s003.pdf]

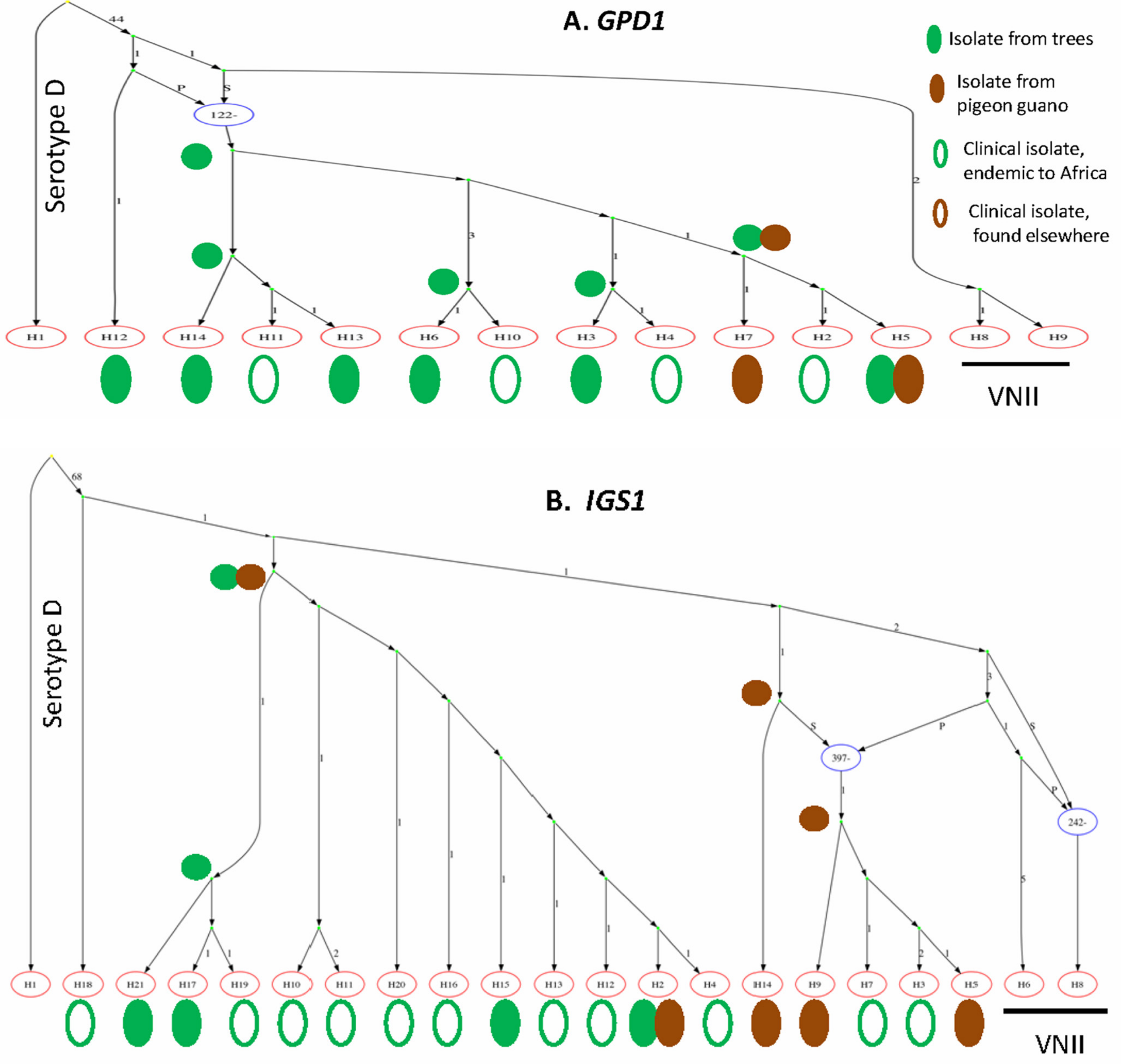

A. GPD1
Isolate from trees
Isolate from pigeon guano
Clinical isolate, endemic to Africa
Clinical isolate, found elsewhere
Serotype D
122-
H1
H12
H14
H11
H13
H6
H10
H3
H4
H7
H2
H5
H8
H9
VNII
B. IGS1
Serotype D
397-
242-
H1
H18
H21
H17
H19
H10
H11
H20
H16
H15
H13
H12
H2
H4
H14
H9
H7
H3
H5
H6
H8
VNII

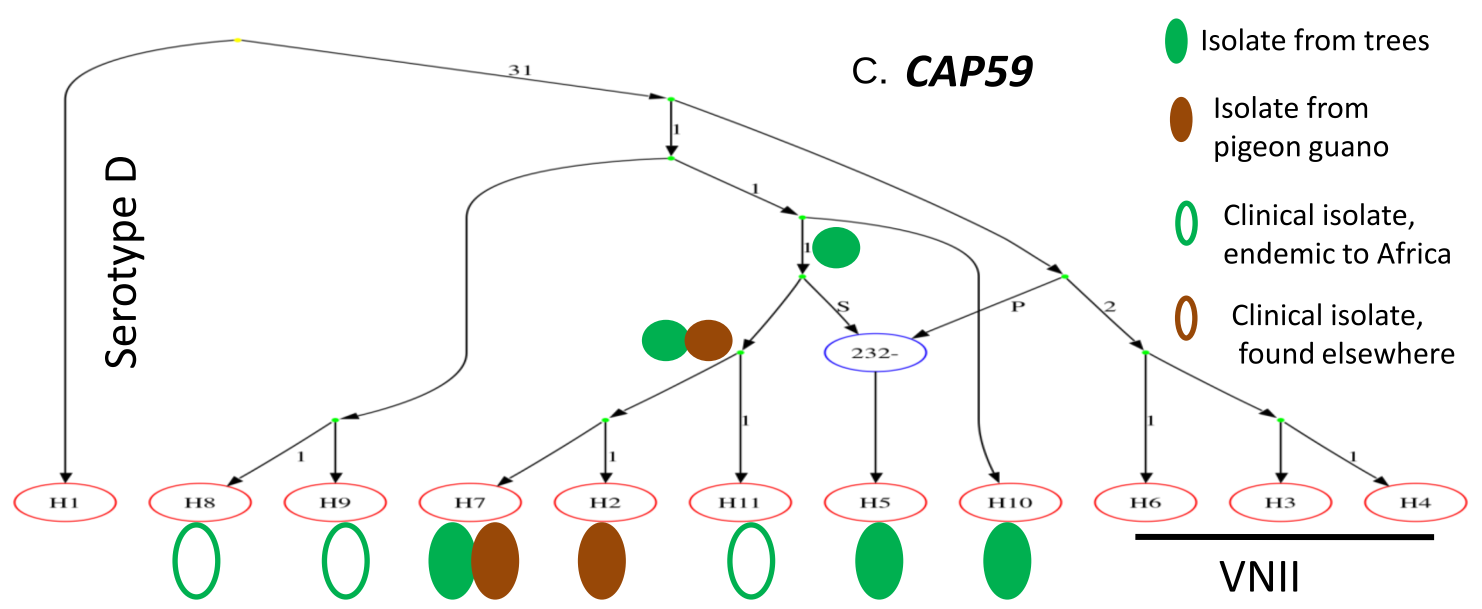

C. CAP59
Isolate from trees
Isolate from pigeon guano
Clinical isolate, endemic to Africa
Clinical isolate, found elsewhere
Serotype D
31
1
1
1
S
P
2
232-
1
1
1
1
1
1
H1
H8
H9
H7
H2
H11
H5
H10
H6
H3
H4
VNII

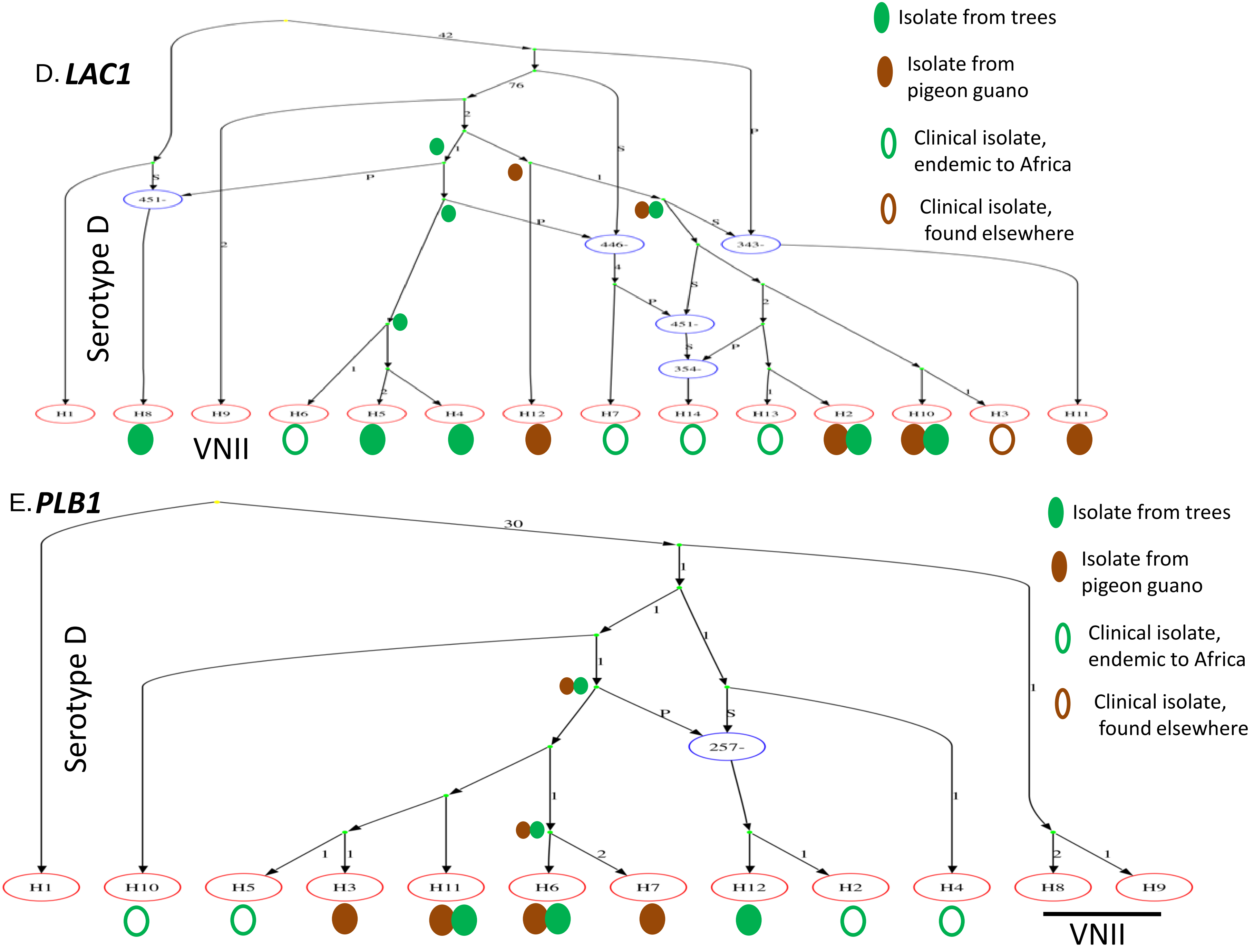
D. LAC1
Isolate from trees
Isolate from pigeon guano
Clinical isolate, endemic to Africa
Clinical isolate, found elsewhere
Serotype D
VNII
42
76
451-
446-
343-
354-
H1
H8
H9
H6
H5
H4
H12
H7
H14
H13
H2
H10
H3
H11
E. PLB1
Isolate from trees
Isolate from pigeon guano
Clinical isolate, endemic to Africa
Clinical isolate, found elsewhere
Serotype D
30
257-
H1
H10
H5
H3
H11
H6
H7
H12
H2
H4
H8
H9
VNII

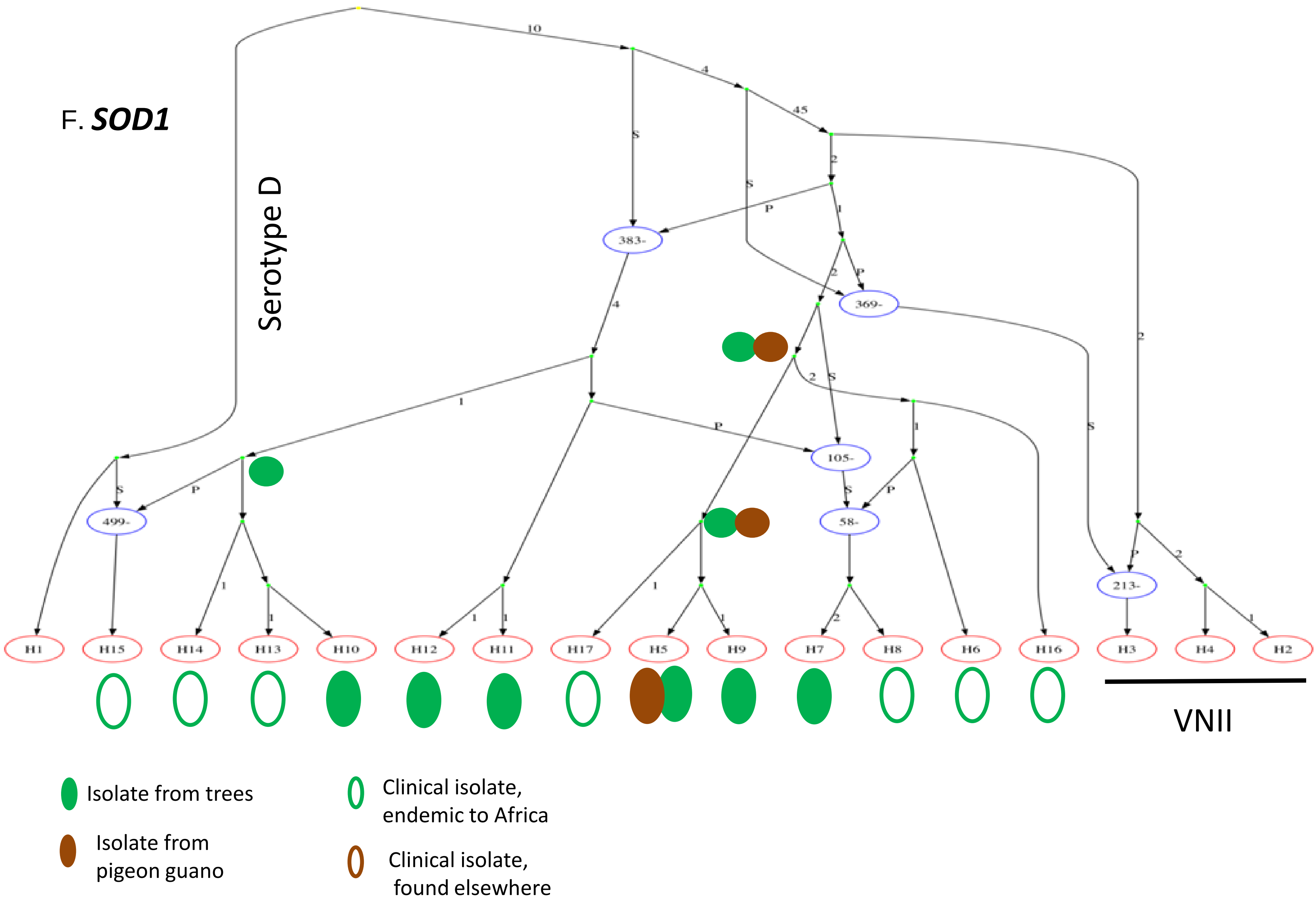

F. SOD1
Serotype D
383-
369-
499-
105-
58-
213-
H1
H15
H14
H13
H10
H12
H11
H17
H5
H9
H7
H8
H6
H16
H3
H4
H2
VNII
Isolate from trees
Isolate from pigeon guano
Clinical isolate, endemic to Africa
Clinical isolate, found elsewhere

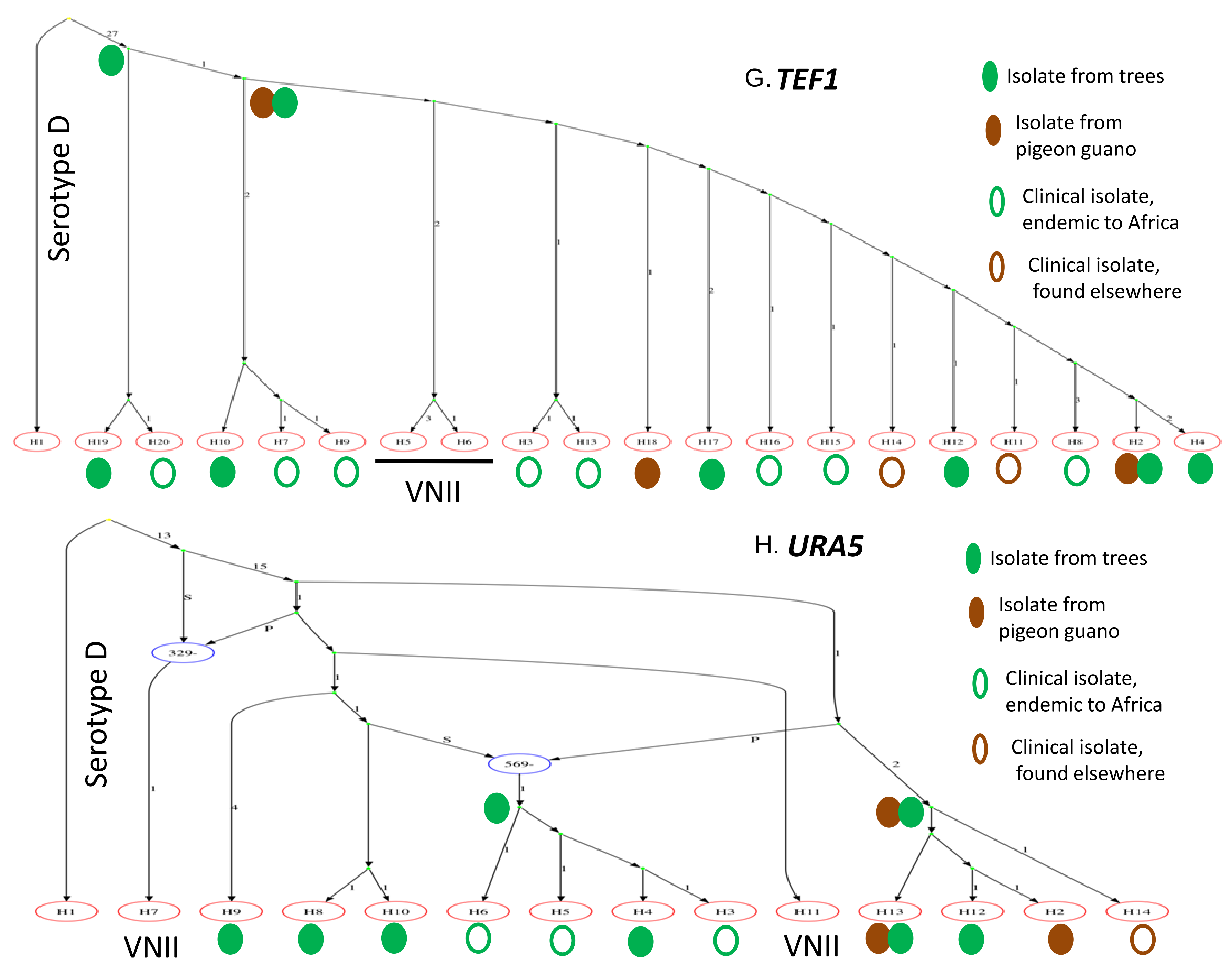

G. TEF1
Isolate from trees
Isolate from pigeon guano
Clinical isolate, endemic to Africa
Clinical isolate, found elsewhere
Serotype D
H1
H19
H20
H10
H7
H9
H5
H6
H3
H13
H18
H17
H16
H15
H14
H12
H11
H8
H2
H4
VNII
H. URA5
Isolate from trees
Isolate from pigeon guano
Clinical isolate, endemic to Africa
Clinical isolate, found elsewhere
Serotype D
329-
569-
H1
H7
H9
H8
H10
H6
H5
H4
H3
H11
H13
H12
H2
H14
VNII
VNII
