# Supplementary material for: Evidence that the Human Pathogenic Fungus Cryptococcus neoformans var. grubii May Have Evolved in Africa
Source: PLoS One. 2011 May 11;6(5):e19688. doi: 10.1371/journal.pone.0019688 (PMC3092753; doi:10.1371/journal.pone.0019688)
Supplement: Table S1 — Environmental samples yielding isolates of Cryptococcus neoformans var. grubii. (PDF) [file pone.0019688.s005.pdf]

Table S1. Environmental samples yielding isolates of *Cryptococcus neoformans* var. *grubii*.

Site	No. of isolates[a]	Location	Environmental source
Tu241	6	Tuli Block, Botswana	soil under *Colophospermum mopane* (mopane tree)
Tu239	20	Tuli Block, Botswana	*C. mopane*
Gb159	10	Gaborone, Botswana	Unidentified tree
Tu259	11	Tuli Block, Botswana	*C. mopane*
Pr284	10	Free State Province, RSA[b]	Pigeon excreta
Pr68	1	Free State Province, RSA	Avian (pigeon and chicken) excreta
Gb118/RTE	50	Gaborone, Botswana	Pigeon excreta
Jo278	17	Johannesburg, RSA	Soil contaminated with avian excreta
D16	25	Durban, RSA	Pigeon excreta
D17	15	Durban, RSA	Pigeon excreta
Tu406	10	Tuli Block, Botswana	*C. mopane*
Tu416	10	Tuli Block, Botswana	*C. mopane*
Tu229	13	Tuli Block, Botswana	*C. mopane*
Tu236	1	Tuli Block, Botswana	Soil under *C. mopane*
Tu369	10	Tuli Block, Botswana	*C. mopane*
Tu372	9	Tuli Block, Botswana	*C. mopane*

Site	No. of isolates[a]	Location	Environmental source
Tu401	10	Tuli Block, Botswana	*C. mopane*
Tu248	2	Tuli Block, Botswana	*Adansonia digitata* (Baobab tree)
Tu360	4	Tuli Block, Botswana	*C. mopane*
Tu422	10	Tuli Block, Botswana	*C. mopane*
Ze90	19	North Western Province, RSA	*Eucalyptus* sp. tree
Ze93	10	North Western Province, RSA	Soil under *Eucalyptus sp.* tree

[a] Total = 273 isolates

[b] RSA, Republic of South Africa
