# Supplementary material for: Evidence that the Human Pathogenic Fungus Cryptococcus neoformans var. grubii May Have Evolved in Africa
Source: PLoS One. 2011 May 11;6(5):e19688. doi: 10.1371/journal.pone.0019688 (PMC3092753; doi:10.1371/journal.pone.0019688)
Supplement: Table S2 — Descriptions of the 142 strains of Cryptococcus neoformans var. grubii used in this study. (PDF) [file pone.0019688.s006.pdf]

Table S2. Descriptions of the 142 strains of *Cryptococcus neoformans* var. *grubii* used in this study.

Strain	Geographic origin	Source	Year	Mating type	VN type	Other strains with the same genotype (countries where the genotype was isolated) [a]	Reference
Bt1	Botswana	CSF [b], HIV+ [c]	1999	α	VNB		(10)
Bt27	Botswana	CSF, HIV+	2000	α	VNB		(10)
Bt24	Botswana	CSF, HIV+	2000	**a**	VNB		(10)
Bt206	Botswana	CSF, HIV+	2002	**a**	VNB		(10)
Bt35	Botswana	CSF, HIV+	2000	α	VNB		(10)
Bt63	Botswana	CSF, HIV+	2000	**a**	VNB		(10)
Bt100	Botswana	CSF, HIV+	2001	α	VNI		(10)
Bt150	Botswana	CSF, HIV+	2001	α	VNI	RSA848, RSA4321, RSA2294 (RSA)	(10)
Bt121	Botswana	CSF, HIV+	2002	α	VNI	RSA2960, RSA5084, RTC5, RTC6 (RSA)	(10)
Bt15	Botswana	CSF, HIV+	2000	α	VNI		(10)
Bt85	Botswana	CSF, HIV+	2001	**a**	VNB		(10)
Bt9	Botswana	CSF, HIV+	1999	α	VNI		(10)
A1-35-8	North Carolina, USA	pigeon excreta	2002	α	VNI	C23, WM148, H99, Arg1366, Mal 120, Fr1 (Argentina, Australia, France, Malawi, USA)	(10)
C23	North Carolina, USA	bronchial wash, HIV- [c]	2001	α	VNI	A1-35-8, WM148, H99, Arg1366, Mal 120, Fr1 (Argentina, Australia, France, Malawi, USA)	(10)
WM148	Australia	CSF	1995	α	VNI	C23, A1-35-8, H99, Arg1366, Mal 120, Fr1 (Argentina, Australia, France, Malawi, USA)	(16)
Br795	Brazil		1998	α	VNI	RTC1, Gb118, RSA1054, Ug2471, In2629, Blg10, Tn10 (Belgium,	(10)

Strain	Geographic origin	Source	Year	Mating type	VN type	Other strains with the same genotype (countries where the genotype was isolated) [a]	Reference
						Botswana, Brazil, India, Tanzania, RSA, Uganda)	
H99	North Carolina, USA	Clinical		α	VNI	C23, WM148, Arg1366, Mal 120, Fr1 (Argentina, Australia, France, Malawi, USA)	(16)
A5-35-17	North Carolina, USA	pigeon excreta	2002	α	VNI	D16-11, C8, Jo278, Jp1086, Mal 212, Bt134, It743, CHC30, Blg7 (Belgium, Botswana, China, Italy, Japan, Malawi, RSA, USA)	(10, 38)
C8	North Carolina, USA	CSF, HIV+	2001	α	VNI	D16-11, A5-35-17, Jo278, Jp1086, Mal 212, Bt134, It743, CHC30, Blg7 (Belgium, Botswana, China, Italy, Japan, Malawi, RSA, USA)	(10, 38)
A2-102-5	Texas, USA	pigeon excreta	2003	α	VNI	D17-1, D17-4, D16-10 (RSA)	(10)
A3-1-1	North Carolina, USA	pigeon excreta	2002	α	VNI	D16-1, RSA1842, RSA2299, Pr284-2, Pr68, It754, Blg12, Fr5 Ug2459 (Belgium, France, Italy, RSA, Uganda)	(10)
A4-1-12	North Carolina, USA	pigeon excreta	2002	α	VNI	Fr4 (France)	(10)
M9	Malawi	CSF, blood, HIV+		α	VNI	In2637 (India)	(10)
Ug2471	Uganda	CSF, HIV+	2001	α	VNI	RTC1, Gb118, RSA1054, In2629, Blg10, Tn10, Br795 (Belgium, Botswana, Brazil, India, Tanzania, RSA)	(10)
Th84	Thailand	blood, HIV+	1997	α	VNI	Ug2463 (Uganda)	(10)
Th104	Thailand	blood, HIV+	1997	α	VNI	RSA2297, Mal 104, Tn148 (Malawi, RSA, Thailand, Tanzania)	(10)
125.91	Tanzania	CSF, HIV+		**a**	VNI		(11, 41)
C26	North Carolina, USA	Blood, HIV+	2001	α	VNI		(10)

Strain	Geographic origin	Source	Year	Mating type	VN type	Other strains with the same genotype (countries where the genotype was isolated) [a]	Reference
C27	North Carolina, USA	CSF, cancer	2001	α	VNI	In2632, Za1345, RSA1105 (DRC [d], India, RSA)	(10)
A4-34-6	North Carolina, USA	pigeon excreta	2003	α	VNI		(10)
8-1	North Carolina, USA	Organ transplant		α	VNII		(14)
C16	North Carolina, USA	sputum, HIV-	2001	α	VNII		(10)
C2	North Carolina, USA	bronchial wash, HIV-	2002	α	VNII		(10)
C44	North Carolina, USA	CSF, HIV-	2002	α	VNII		(10)
A7-35-23	North Carolina, USA	pigeon excreta	2003	α	VNII		(10)
C45	North Carolina, USA	sputum, HIV-	2001	α	VNII		(10)
WM626	Australia	CSF	1995	α	VNII		(16)
RSA1684	Limpopo Pr., RSA [d]	Clinical	2006	α	VNII	RSA1831 (RSA)	This study
RSA1831	Limpopo Pr., RSA	Clinical	2006	α	VNII	RSA1684 (RSA)	This study
RSA1054	Limpopo Pr., RSA	Clinical	2006	α	VNI	RTC9, Gb118, RTE-ALL, Ug2471, In2629, Blg10, Tn10, Br795 (Belgium, Botswana, Brazil, India, Tanzania, RSA, Uganda)	This study
RSA1105	Limpopo Pr., RSA	Clinical	2006	α	VNI	C27, In2632, Za1345 (DRC, India, RSA)	This study
RSA1271	Limpopo Pr. [d], RSA	Clinical	2006	α	VNI	RSA1442, RSA3796 (RSA)	This study
RSA1284	Limpopo Pr., RSA	Clinical	2006	α	VNI		This study
RSA1332	Limpopo Pr., RSA	Clinical	2006	α	VNI		This study
RSA1354	Limpopo Pr., RSA	Clinical	2006	α	VNI		This study
RSA1442	Limpopo Pr., RSA	Clinical	2006	α	VNI	RSA1271, RSA3796 (RSA)	This study

Strain	Geographic origin	Source	Year	Mating type	VN type	Other strains with the same genotype (countries where the genotype was isolated) [a]	Reference
RSA1691	Limpopo Pr., RSA	Clinical	2006	α	VNI		This study
RSA1842	Limpopo Pr., RSA	Clinical	2006	α	VNI	A3-1-1, D16-1, RSA2299, RSA480, Pr284-2, Pr68-1, It754, Blg12, Fr5 (Belgium, France, Italy, RSA, Uganda, USA)	This study
RSA2297	Limpopo Pr., RSA	Clinical	2006	α	VNI	Th104, Mal 104, Tn148 (Malawi, RSA, Tanzania, Thailand)	This study
RSA2299	Limpopo Pr., RSA	Clinical	2006	α	VNI	A3-1-1, D16-1, RSA1842, RSA480, Pr284-2, Pr68-1, It754, Blg12, Fr5 (Belgium, France, Italy, RSA, Uganda, USA)	This study
RSA263	Limpopo Pr., RSA	Clinical	2006	α	VNI		This study
RSA2960	Limpopo Pr., RSA	Clinical	2006	α	VNI	Bt121, RSA5084, RTC5, RTC6 (Botswana, RSA)	This study
RSA3751	Limpopo Pr., RSA	Clinical	2006	α	VNI		This study
RSA3796	Limpopo Pr., RSA	Clinical	2006	α	VNI	RSA1271, RSA1442 (RSA)	This study
RSA4068	Limpopo Pr., RSA	Clinical	2006	α	VNI		This study
RSA4389	Limpopo Pr., RSA	Clinical	2006	α	VNI		This study
RSA480	Limpopo Pr., RSA	Clinical	2006	α	VNI	A3-1-1, D16-1, RSA1842, RSA2299, Pr284-2, Pr68-1, It754, Blg12, Fr5 (Belgium, France, Italy, RSA, Uganda, USA)	This study
RSA5084	Limpopo Pr., RSA	Clinical	2006	α	VNI	Bt121, RSA2960, RTC5, RTC6 (Botswana, RSA)	This study
RSA730	Limpopo Pr., RSA	Clinical	2006	α	VNI	A5-35-17, C8, D16-11, Jo278-1, Jp1086, Mal 212, It743, CHC30, Blg7 (Belgium, Botswana, China, Italy, Japan, Malawi, RSA, USA)	This study
RSA731	Limpopo Pr., RSA	Clinical	2006	α	VNI	A5-35-17, C8, D16-11, Jo278-1, Jp1086, Mal 212, It743, CHC30, Blg7 (Belgium, Botswana, China, Italy, Japan, Malawi, RSA, USA)	This study
RSA1343	Limpopo Pr., RSA	Clinical	2006	α	VNI		This study

Strain	Geographic origin	Source	Year	Mating type	VN type	Other strains with the same genotype (countries where the genotype was isolated) [a]	Reference
RSA3042	Limpopo Pr., RSA	Clinical	2006	α	VNI		This study
RSA4129	Limpopo Pr., RSA	Clinical	2006	α	VNI		This study
RSA1082	Limpopo Pr., RSA	Clinical	2006	α	VNB		This study
RSA6618	Limpopo Pr., RSA	Clinical	2006	α	VNB		This study
RSA3768	Limpopo Pr., RSA	Clinical	2006	a	VNB		This study
RSA116	Limpopo Pr., RSA	Clinical	2006	α	VNI		This study
RSA209	Limpopo Pr., RSA	Clinical	2006	α	VNI		This study
RSA2294	Limpopo Pr., RSA	Clinical	2006	α	VNI	Bt150, RSA848, RSA4321 (Botswana, RSA)	This study
RSA4321	Limpopo Pr., RSA	Clinical	2006	α	VNI	Bt150, RSA848, RSA2294 (Botswana, RSA)	This study
RSA848	Limpopo Pr., RSA	Clinical	2006	α	VNI	Bt150, RSA2294, RSA4321 (Botswana, RSA)	This study
RSA2296	Limpopo Pr., RSA	Clinical	2006	α	VNI		This study
RSA314	Limpopo Pr., RSA	Clinical	2006	α	VNI		This study
RSA3573	Limpopo Pr., RSA	Clinical	2006	α	VNB		This study
RSA5433	Limpopo Pr., RSA	Clinical	2006	α	VNB		This study
RSA756	Limpopo Pr., RSA	Clinical	2006	α	VNB		This study
RSA3144	Limpopo Pr., RSA	Clinical	2006	α	VNB		This study
RTC1	Botswana	Clinical	2007	α	VNI	Br795, Ug2458, Gb118, Blg10, Tn10, Bt, In2629, RSA1054 (Belgium, Botswana, Brazil, India, RSA, Tanzania, Uganda)	This study
RTC2	Botswana	Clinical	2007	α	VNB	RTC4 (Botswana)	This study
RTC3	Botswana	Clinical	2007	α	VNB		This study

Strain	Geographic origin	Source	Year	Mating type	VN type	Other strains with the same genotype (countries where the genotype was isolated) [a]	Reference
RTC4	Botswana	Clinical	2007	α	VNB	RTC2 (Botswana)	This study
RTC5	Botswana	Clinical	2007	α	VNI	Bt121, RSA2960, RSA5084, RTC6 (Botswana, RSA)	This study
RTC6	Botswana	Clinical	2007	α	VNI	Bt121, RSA2960, RSA5084, RTC5 (Botswana, RSA)	This study
RTC9	Botswana	Clinical	2007	α	VNI	RSA1054, Gb118, RTE-ALL, Ug2471, In2629, Blg10, Tn10, Br795 (Belgium, Botswana, Brazil, India, Tanzania, RSA, Uganda)	This study
D16-1	Durban, RSA	pigeon excreta	2007	α	VNI	A3-1-1, D16-1, RSA2299, RSA480, Pr284-2, Pr68-1, It754, Blg12, Fr5 (Belgium, France, Italy, RSA, USA)	This study
D16-2	Durban, RSA	pigeon excreta	2007	α	VNI	D16-4, D16-16, D16-7, D17-2, D17-5, D17-7 (RSA)	This study
D16-3	Durban, RSA	pigeon excreta	2007	α	VNI	A3-1-1, D16-14, D16-1, RSA2299, RSA480, Pr284-2, Pr68-1, It754, Blg12, Fr5 (Belgium, France, Italy, RSA, USA)	This study
D16-4	Durban, RSA	pigeon excreta	2007	α	VNI	D16-2, D16-16, D16-7, D17-2, D17-5, D17-7 (RSA)	This study
D16-5	Durban, RSA	pigeon excreta	2007	α	VNI		This study
D16-6	Durban, RSA	pigeon excreta	2007	α	VNI	A5-35-17, C8, Jo278, Jp1086, Mal 212, Bt134, It743, Blg7, CHC30 (Belgium, Botswana, China, Italy, Japan, Malawi, RSA, USA)	This study
D16-7	Durban, RSA	pigeon excreta	2007	α	VNI	D16-2, D16-16, D16-4, D17-2, D17-5, D17-7 (RSA)	This study
D16-9	Durban, RSA	pigeon excreta	2007	α	VNI	A5-35-17, C8, Jo278, Jp1086, Mal 212, Bt134, It743, CHC30, Blg7 (Belgium, Botswana, China, Italy, Japan, Malawi, RSA, USA)	This study
D16-10	Durban, RSA	pigeon excreta	2007	α	VNI	A2-102-5, D17-1, D17-4 (RSA, USA)	This study
D16-11	Durban, RSA	pigeon excreta	2007	α	VNI	A5-35-17, C8, Jo278, Jp1086, Mal 212, Bt134, It743, Blg7, CHC30 (Belgium, Botswana, China, Italy, Japan, Malawi, RSA, USA)	This study

Strain	Geographic origin	Source	Year	Mating type	VN type	Other strains with the same genotype (countries where the genotype was isolated) [a]	Reference
D16-12	Durban, RSA	pigeon excreta	2007	α	VNI	A5-35-17, C8, Jo278, Jp1086, Mal 212, Bt134, It743, Blg7, CHC30 (Belgium, Botswana, China, Italy, Japan, Malawi, RSA, USA)	This study
D16-13	Durban, RSA	pigeon excreta	2007	α	VNI	A5-35-17, C8, Jo278, Jp1086, Mal 212, Bt134, It743, Blg7, CHC30 (Belgium, Botswana, China, Italy, Japan, Malawi, RSA, USA)	This study
D16-14	Durban, RSA	pigeon excreta	2007	α	VNI	A3-1-1, D16-1, RSA2299, RSA480, Pr284-2, Pr68, it754, Blg12 (Belgium France, Italy, RSA, Uganda, USA)	This study
D16-16	Durban, RSA	pigeon excreta	2007	α	VNI	D16-2, D16-4, D16-7, D17-2, D17-5, D17-7 (RSA)	This study
D17-1	Durban, RSA	pigeon excreta	2007	α	VNI	A2-102-5, D16-10, D17-4 (RSA, USA)	This study
D17-2	Durban, RSA	pigeon excreta	2007	α	VNI	D16-2, D16-16, D16-7, D17-4, D17-5, D17-7 (RSA)	This study
D17-3	Durban, RSA	pigeon excreta	2007	α	VNI	A5-35-17, C8, Jo278, Jp1086, Mal 212, Bt134, It743, Blg7, CHC30 (Belgium, Botswana, China, Italy, Japan, Malawi, RSA, USA)	This study
D17-4	Durban, RSA	pigeon excreta	2007	α	VNI	A2-102-5, D16-10, D17-1 (RSA, USA)	This study
D17-5	Durban, RSA	pigeon excreta	2007	α	VNI	D16-2, D16-16, D16-7, D17-4, D17-5, D17-2, D17-7 (RSA)	This study
D17-7	Durban, RSA	pigeon excreta	2007	α	VNI	D16-2, D16-16, D16-7, D17-4, D17-5, D17-2, D17-7 (RSA)	This study
Gb118-1	Gaborone, Botswana	pigeon excreta	2007	α	VNI	RSA1054, RTC9, RTE-ALL, Ug2471, In2629, Blg10, Br795, Tn10 (Belgium, Botswana, Brazil, India, Tanzania, RSA, Uganda)	This study
Gb118-2	Gaborone, Botswana	pigeon excreta	2007	α	VNI	RSA1054, RTC9, RTE-ALL, Ug2471, In2629, Blg10, Br795, Tn10 (Belgium, Botswana, Brazil, India, Tanzania, RSA, Uganda)	This study
Gb159-1	Gaborone, Botswana	bark, unidentified tree	2007	α	VNI	Gb159-2 (Botswana)	This study

Strain	Geographic origin	Source	Year	Mating type	VN type	Other strains with the same genotype (countries where the genotype was isolated) [a]	Reference
Gb159-2	Gaborone, Botswana	bark, unidentified tree	2007	α	VNI	Gb159-1 (Botswana)	This study
Jo278-1	Johannesburg, RSA	soil contaminated with avian excreta	2007	α	VNI	A5-35-17, C8, Jp1086, Mal 212, Bt134, It743, CHC30,Jo278-2 (Belgium Botswana, China, Italy, Japan, Malawi, RSA, USA)	This study
Jo278-2	Johannesburg, RSA	soil contaminated with avian excreta	2007	α	VNI	A5-35-17, C8, Jp1086, Mal 212, Bt134, It743, CHC30, Jo278-1 (Belgium Botswana, China, Italy, Japan, Malawi, RSA, USA)	This study
Pr68-1	Parys, RSA	pigeon excreta	2007	α	VNI	A3-1-1, D16-1, RSA2299, RSA480, Pr284-2 , It754, Blg12, Fr5 (Belgium, France, Italy, RSA, Uganda, USA)	This study
Pr284-1	Parys, RSA	pigeon excreta	2007	α	VNI		This study
Pr284-2	Parys, RSA	pigeon excreta	2007	α	VNI	A3-1-1, D16-1, RSA2299, RSA480, Pr68-1, It754, Blg12, Fr5 (Belgium, France, Italy, RSA, Uganda, USA)	This study
RTE-ALL	Gaborone, Botswana	pigeon excreta	2007	α	VNI	RSA1054, RTC9, RTE-ALL, Ug2471, In2629, Blg10, Br795, Tn10 (Belgium, Botswana, Brazil, India, Tanzania, RSA, Uganda)	This study
Tu229-1	Tuli block, Botswana	Mopane bark	2007	α	VNB	Tu229-6, Tu236-1 (Botswana)	This study
Tu229-6	Tuli block, Botswana	Mopane bark	2007	α	VNB	Tu229-1, Tu236-1 (Botswana)	This study
Tu236-1	Tuli block, Botswana	soil under Mopane tree	2007	α	VNB	Tu229-1, Tu229-6 (Botswana)	This study
Tu239-1	Tuli block, Botswana	Mopane bark	2007	α	VNI	Tu-239-2, Tu241-1, Tu241-2 (Botswana)	This study
Tu239-2	Tuli block, Botswana	Mopane bark	2007	α	VNI	Tu-239-1, Tu241-1, Tu241-2 (Botswana)	This study
Tu241-1	Tuli block, Botswana	soil under Mopane tree	2007	α	VNI	Tu-239-1, Tu239-2, Tu241-2 (Botswana)	This study

Strain	Geographic origin	Source	Year	Mating type	VN type	Other strains with the same genotype (countries where the genotype was isolated) [a]	Reference
Tu241-2	Tuli block, Botswana	soil under Mopane tree	2007	α	VNI	Tu-239-1, Tu239-2, Tu241-1 (Botswana)	This study
Tu248-1	Tuli block, Botswana	Baobab	2007	α	VNB	Tu248-2 (Botswana)	This study
Tu248-2	Tuli block, Botswana	Baobab	2007	α	VNB	Tu248-1 (Botswana)	This study
Tu259-1	Tuli block, Botswana	Mopane bark	2007	α	VNI	Tu259-2 (Botswana)	This study
Tu259-2	Tuli block, Botswana	Mopane bark	2007	α	VNI	Tu259-1 (Botswana)	This study
Tu360-1	Tuli block, Botswana	Mopane bark	2007	α	VNB	Tu360-2 (Botswana)	This study
Tu360-2	Tuli block, Botswana	Mopane bark	2007	α	VNB	Tu360-1 (Botswana)	This study
Tu369-1	Tuli block, Botswana	Mopane bark	2007	α	VNB	Tu369-2, Tu372-1, Tu372-2 (Botswana)	This study
Tu369-2	Tuli block, Botswana	Mopane bark	2007	α	VNB	Tu369-1, Tu372-1, Tu372-2 (Botswana)	This study
Tu372-1	Tuli block, Botswana	Mopane bark	2007	α	VNB	Tu369-1, Tu369-2, Tu372-1 (Botswana)	This study
Tu372-2	Tuli block, Botswana	Mopane bark	2007	α	VNB	Tu369-1, Tu369-2, Tu372-2 (Botswana)	This study
Tu401-1	Tuli block, Botswana	Mopane bark	2007	α	VNB		This study
Tu406-1	Tuli block, Botswana	Mopane bark	2007	α	VNB	Tu406-2 (Botswana)	This study
Tu406-2	Tuli block, Botswana	Mopane bark	2007	α	VNB	Tu406-2 (Botswana)	This study
Tu416-1	Tuli block, Botswana	Mopane bark	2007	α	VNB	Tu416-2 (Botswana)	This study
Tu416-2	Tuli block, Botswana	Mopane bark	2007	α	VNB	Tu416-1 (Botswana)	This study
Tu422-1	Tuli block, Botswana	Mopane bark	2007	a	VNB	Tu422-2 (Botswana)	This study
Tu422-2	Tuli block, Botswana	Mopane bark	2007	a	VNB	Tu422-2 (Botswana)	This study
Ze90-1	Zeerust, RSA	Eucalyptus bark	2007	α	VNB	Ze90-2, Ze93-1, Ze93-2 (RSA)	This study

Strain	Geographic origin	Source	Year	Mating type	VN type	Other strains with the same genotype (countries where the genotype was isolated) [a]	Reference
Ze90-2	Zeerust, RSA	Eucalyptus bark	2007	α	VNB	Ze90-1, Ze93-1, Ze93-2 (RSA)	This study
Ze93-1	Zeerust, RSA	soil under Eucalyptus tree	2007	α	VNB	Ze90-1, Ze90-2, Ze93-2 (RSA)	This study
Ze93-2	Zeerust, RSA	soil under Eucalyptus tree	2007	α	VNB	Ze90-1, Ze90-2, Ze93-1 (RSA)	This study

[a] Other strains with the same genotype. Because of space considerations, only representative strains are included. A complete list of each global strain and its origin is provided in ref. 10 and 38.

[b] CSF, cerebrospinal fluid.

[c] HIV+, HIV-infected; HIV-, non-HIV-infected.

[d] RSA, Republic of South Africa; DRC, Democratic Republic of the Congo, Pr., Province.
