# Supplementary material for: Evidence that the Human Pathogenic Fungus Cryptococcus neoformans var. grubii May Have Evolved in Africa
Source: PLoS One. 2011 May 11;6(5):e19688. doi: 10.1371/journal.pone.0019688 (PMC3092753; doi:10.1371/journal.pone.0019688)
Supplement: Table S3 — Indices of association (IA) among the loci in subpopulations of C. neoformans var. grubii. (PDF) [file pone.0019688.s007.pdf]

Table S3. Indices of association (I_A) among the loci in subpopulations of *C. neoformans* var *grubii*.

Subpopulation	Source	Number of genotypes	I_A	*P*
Native African (all genotypes)[a]	native trees	12	0.45	0.01
**Native African (VNB strains only)**	native trees[b]	8	**0.3**[d]	**0.08**
**Native African (VNB strains only)**	clinical	8	**-0.09**	**0.6**
**Native African (VNB strains only)**	native trees and clinical	16	**-0.09**	**0.7**
**Native African (VNI strains only)**[c]	native trees and clinical	24	**0.08**	**0.3**
Native African (VNI strains only)	native trees	3	NA[e]	NA
Global (VNI strains only)	pigeon excreta and clinical	12	0.6	0.009

[a] Unique genotypes of all strains isolated from trees are included.

[b] Only arboreal genotypes from Botswana were included. (We excluded the Ze90/Ze93 genotype isolated from a *Eucalyptus* tree in western RSA.)

[c] Only unique genotypes from Botswana and RSA are included.

[d] Values of I_A for which the null hypothesis of recombination cannot be rejected are bolded.

[e] The total number of unique genotypes is too low to calculate an I_A value.
