# Supplementary material for: Evidence that the Human Pathogenic Fungus Cryptococcus neoformans var. grubii May Have Evolved in Africa
Source: PLoS One. 2011 May 11;6(5):e19688. doi: 10.1371/journal.pone.0019688 (PMC3092753; doi:10.1371/journal.pone.0019688)
Supplement: Table S4 — Results of Templeton (T) and Kishino-Hasegawa (K-H) tests for conflict among phylogenetic topologies of the gene genealogies at each locus. (PDF) [file pone.0019688.s008.pdf]

Table S4. Results of Templeton (T) and Kishino-Hasegawa (K-H) tests for conflict among phylogenetic topologies of the gene genealogies at each locus. Numbers listed are the T and K-H probabilities of rejecting the null hypothesis that there is no difference between the maximum parsimony tree topology of a given locus (columns) and its data constrained by the maximum parsimony topology of each of the other seven loci. *p* values ≤0.05 are in **bold.**

Locus	*CAP59*		*GPD1*		IGS1		*LAC1*		*PLB1*		*SOD1*		*TEF1*		*URA5*	
	T	K-H	T	K-H	T	K-H	T	K-H	T	K-H	T	K-H	T	K-H	T	K-H
**A.** Results when the calculations included data from both African (VNB+VNI-Botswana) and global (VNI+VNII) strains																
CAP59	-	-	**0.007**	**0.02**	**0.0009**	**0.0007**	**0.007**	**0.01**	**0.04**	0.06	**0.0004**	**0.0003**	**0.005**	**0.03**	**<0.0001**	**<0.0001**
GPD1	0.07	0.07	-	-	**0.0009**	**0.0007**	**0.007**	**0.02**	**0.04**	**0.04**	**0.0004**	**0.001**	**0.01**	**0.04**	**<0.0001**	**<0.0001**
IGS1	**0.04**	**0.05**	**0.003**	**0.01**	-	-	**0.03**	**0.03**	0.06	**0.04**	**0.0004**	**0.0003**	**0.005**	**0.03**	**<0.0001**	**<0.0001**
LAC1	**0.04**	0.07	**0.003**	**0.01**	**0.002**	**0.002**	-	-	**0.04**	**0.04**	**0.0004**	**0.0003**	**0.005**	**0.02**	**<0.0001**	**<0.0001**
PLB1	**0.04**	0.11	**0.003**	**0.01**	**0.0009**	**0.002**	**0.007**	**0.01**	-	-	**0.02**	**0.02**	**0.005**	**0.03**	**<0.0001**	**<0.0001**
SOD1	**0.04**	**0.05**	**0.005**	**0.02**	**0.0009**	**0.005**	**0.007**	**0.01**	0.1	0.12	-	-	**0.01**	0.08	**<0.0001**	**<0.0001**
TEF1	**0.04**	**0.05**	**0.005**	**0.03**	**0.0009**	**0.002**	**0.007**	**0**	**0.04**	0.06	**0.0004**	**0.0003**	-	-	**<0.0001**	**<0.0001**
URA5	**0.04**	0.06	**0.01**	**0.04**	**0.0009**	**0.004**	**0.01**	**0.02**	0.1	0.11	**0.02**	**0.04**	**0.005**	**0.05**	-	-
**B.** Results when the calculations included data only from global (VNI+VNII) strains																
CAP59	-	-	0.1	0.07	**0.005**	**0.005**	0.07	0.08	0.3	0.3	0.08	0.08	0.2	0.2	0.3	0.3
GPD1	0.3	0.3	-	-	**0.005**	**0.002**	0.07	0.07	0.2	0.2	1	1	0.2	0.2	0.3	0.3
IGS1	0.3	0.3	0.06	0.1	-	-	0.07	0.06	0.2	0.2	0.08	0.08	0.2	0.2	0.3	0.3
LAC1	0.3	0.3	0.06	0.1	**0.004**	**0.002**	-	-	0.2	0.2	0.08	0.08	0.2	0.2	0.3	0.3
PLB1	1	1	0.06	0.1	**0.005**	**0.005**	0.07	0.08	-	-	0.08	0.08	0.2	0.2	0.3	0.3
SOD1	0.3	0.3	0.1	0.1	**0.005**	**0.01**	0.07	0.07	0.2	0.2	-	-	0.2	0.2	0.3	0.3
TEF1	0.3	0.3	0.07	0.1	**0.005**	**0.005**	0.07	**0.04**	0.2	0.2	0.08	0.08	-	-	0.3	0.3
URA5	0.3	0.3	0.07	0.1	**0.005**	**0.006**	0.06	0.06	0.2	0.2	0.08	0.08	0.2	0.2	-	-
