# Supplementary material for: Evidence that the Human Pathogenic Fungus Cryptococcus neoformans var. grubii May Have Evolved in Africa
Source: PLoS One. 2011 May 11;6(5):e19688. doi: 10.1371/journal.pone.0019688 (PMC3092753; doi:10.1371/journal.pone.0019688)
Supplement: Table S5 — MLST primers and PCR conditions used in this study. (PDF) [file pone.0019688.s009.pdf]

Table S5. MLST Primers and PCR conditions.

Locus	Primer sequence	Tm, °C	Product size, bp	PCR Conditions
GPD-f	5'-ATG GTC GTC AAG GTT GGA AT	55.5	599	12 cycles 62-56°C step-down 2°C every 2 cycles followed by 25 cycles at 56°C
GPD-r	5'-GTA TTC GGC ACC AGC CTC A	58.1		
PLB1-f	5'-CTT CAG GCG GAG AGA GGT TT	57.7	674	
PLB1-r	5'-GAT TTG GCG TTG GTT TCA GT	55.2		
SOD1-f	5'-TCT AAT CGA AAT GGT CAA GG	50.7	680	
SOD1-r	5'-CGC AGC TGT TCG TCT GGA TA	58.1		
TEF1-f	5'-AAT CGT CAA GGA GAC CAA CG	55.9	844	10 cycles 62-52°C step-down 1°C every 1 cycle followed by 25 cycles at 52°C
TEF1-r	5'-CGT CAC CAG ACT TGA CGA AC	56.5		
LAC-f	5'-GGC GAT ACT ATT ATC GTA	45.6	586	35 cycles 50°C
LAC-r	5'-TTC TGG AGT GGC TAG AGC	54.1		
IGS1-f	5'-ATC CTT TGC AGA CGA CTT GA	55.3	790	30 cycles 56°C
IGS1-r	5'-GTG ATC AGT GCA TTG CAT GA	54.7		
URA5-f	5'-ATG TCC TCC CAA GCC CTC GAC	61.4	601	35 cycles 63°C
URA5-r	5'-TTA AGA CCT CTG AAC ACC GTA CTC	55.9		
CAP59-f [a]	5'-CTC TAC GTC GAG CAA GTC AAG	55.1	559	35 cycles, 54°C
CAP59-r	5'-TCC GCT GCA CAA GTG ATA CCC	59.7		

[a] *CAP59* locus from the consensus MLST project [16]
